# Quantifying US air pollution policy: How political and regional factors influence pollutant mitigation

**DOI:** 10.1093/pnasnexus/pgae199

**Published:** 2024-05-17

**Authors:** Guoxing Zhang, Zhanglei Chen, Jiexun Li, Bin Su, Yang Gao, Lean Yu

**Affiliations:** School of Management, Lanzhou University, Lanzhou 730000, China; School of Management, Lanzhou University, Lanzhou 730000, China; Department of Decision Sciences, Western Washington University, Bellingham, WA 98225, USA; Energy Studies Institute, National University of Singapore, 119620, Singapore, Singapore; Department of Industrial Systems Engineering and Management, National University of Singapore, 117576, Singapore, Singapore; School of Management, Lanzhou University, Lanzhou 730000, China; Business School, Sichuan University, Chengdu 610065, China

**Keywords:** Clean Air Act, policy intensity, political polarization, natural language processing, policy effectiveness

## Abstract

Air pollution control in the United States has evolved into a comprehensive policy system spanning from the federal to the state level over time. A unified quantitative analysis of policy intensity can shed light on the policy evolution across different levels, the influence of partisan and regional factors on policy, and the relationships with emissions of major pollutants. By harnessing the policy text of the Clean Air Act (CAA) at the federal level and State Implementation Plans (SIPs) at the state governments (1955–2020), we deployed a Natural Language Processing approach to define a policy intensity index to systematically quantify the US air policy landscape. Our findings highlight that the 1970 CAA amendment carries the most vigorous intensity as it established a holistic control system for the first time. Subsequent years witnessed a general trend of partisan polarization, eventually leading to a graduate convergence between red and blue states. Blue states demonstrated a closer alignment with federal directives and a superior efficacy in pollutant reduction. Regionally, the Northeast displays the highest overall policy intensity, and the West exhibits the highest coordination with the federal benchmarks, making these regions outperform others in air pollution control. Our study not only discusses policy implications for air pollutant reductions considering partisan and regional differences but also provides a novel measurement tool to quantify policies for assessing disparities and synergies.

**Significance Statement**

The Clean Air Act stands as a pivotal legislation in US history. Yet, the absence of comprehensive measures has impeded the assessment of policy efficacy. Via our novel framework of data preprocessing, indicator development, and application, we combine methods from Natural Language Processing, Maharaj Distance, multidimensional scaling, and econometrics. This enables the transformation of unstructured policy texts into quantifiable policy intensity indicators. These indicators can adeptly characterize crucial policy shifts, political polarization, and regional variances. Our study evaluates the effects of these policies and further offers recommendations. Importantly, our innovative measurement toolkit is applicable to various types of policy texts, uncovering the impacts of multifaceted factors on policy.

## Introduction

The Clean Air Act (CAA) is a top-down national act of the US's efforts to regulate major air pollutants. Through a cooperative model, the federal government takes charge of coordination and regulation, while states exercise considerable discretion in their governance (1, 2). This has led in remarkable results in air pollutant control (3, 4), saving tens of thousands more lives than initially projected (5). Regional disparities, such as party preference, economic development, geographical conditions, and industrial structure, influence the process of policy making and impact policy efficacy. A comprehensive and chronological study of policy trajectories can shed light on the potency of these instruments (6).

Existing studies on environmental policy evolution mainly focus on specific policies such as solar photovoltaic (7), biofuel (8), and wind energy pricing policies (9). For instance, Schmalensee and Stavins (10) examined the two major amendments of the CAA in 1970 and 1990, driving key insights that inform this article. When it comes to analyzing large amounts of policy documents that span decades, conventional methods struggle to extract multidimensional insights from such volumes. Furthermore, manual analysis often succumbs to subjective or political biases.

Partisan and regional factors play a pivotal role in shaping policy. Many studies highlight the influence of partisan polarization on people's attitudes and behaviors toward climate policies (11, 12). In general, Democrats demonstrated more concerns for the environment than Republicans, leading to a de facto partisan divide (13). While conservatives (often aligned with Republicans) often exhibit hesitance towards endorsing climate action (14), their support for environmental policy tends to be driven by economic, security, or health motivations (12, 15, 16). When air pollution is framed in the context of climate change rather than public health, liberals (typically Democrats) are more inclined to support it (15), due to their pronounced concerns about global climate change (17), pursuit of environmental quality (18), and identification with environmentalism (19).

Region-specific policies are an essential part of the policy framework of many countries (20). Local governments often possess more information and can swiftly address regional demands (21, 22), and yet these policies vary considerably due to regional differences (23). For example, the climate policies in California and Arizona in 2020 exhibited a difference of 1.76 standard deviations (24). While most US states have a renewable portfolio standard, only South Carolina enforces a state-level renewable energy mandate (25), due to this region's limited renewable energy resources and concerns that mandatory regulations might decrease affordability and reliability of electricity (26). The impact of party affiliation and regional distinctions on policy making, especially in the context of CAA, remains underexplored. Moreover, few existing studies have sought to quantify these disparities attributes using policy texts.

The enforcement of CAA, in conjunction with other regulations and policies, has substantially enhanced air quality in the United States (27). For instance, studies have found that policies promulgated by the North American Emission Control District and the California Air Resources Board, targeting sulfur levels in marine fuel oils, have significantly reduced vanadium concentrations linked to fuel oil combustion, observed at coastal monitoring sites in the San Francisco Bay Area and across the United States (28, 29). Furthermore, a risk assessment model confirmed that the NC Clean Smokestacks Act enacted in North Carolina has significantly reduced local SO_2_ and particulate sulfate concentrations (30). A cross-sectional comparison of 10 different state-level policies aimed at curbing fossil fuel combustion to reduce carbon emissions found that carbon pricing policies outperform other policies in efficacy (31). However, existing studies predominantly focus on the outcomes of region-specific policies. There is an absence of a systematic assessment of policies widely implemented in the nation, such as the CAA, for a cross-regional comparison of emission reductions.

**Fig. 1.**
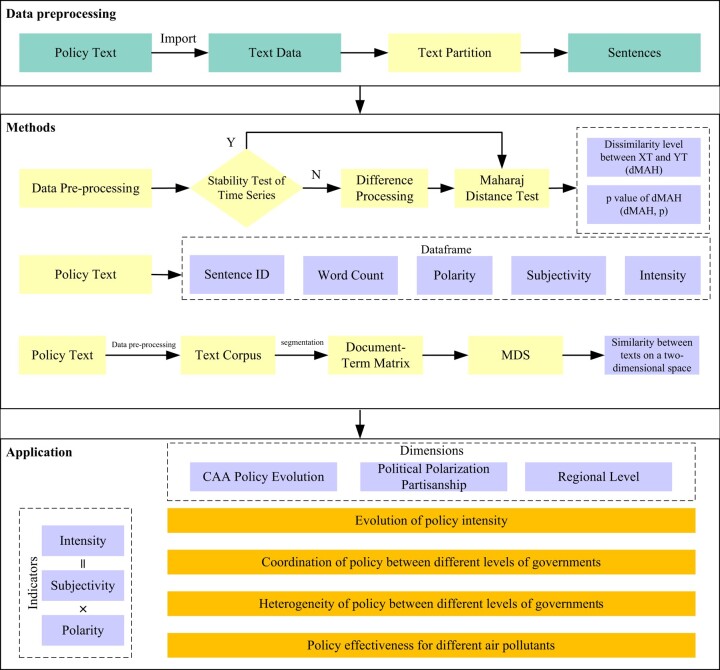
Text preprocessing and analysis procedures.

We seek to address the research gaps by investigating the following questions: How has the CAA, under the influence of major policy shifts, political polarization, and regional disparities, evolved over a long time? And how do these factors shape the policy efficacy? As shown in Fig. 1, this research comprehensively measures and analyzes the policy intensity of the CAA of the federal government and SIPs of different states over the past five decades. Using Natural Language Processing (NLP), we quantitatively analyze vast policy texts and integrate policies enacted by different levels of government into a unified evaluation system. Furthermore, with the Maharaj Distance and MDS method, we illustrate policy coordination and disparities from different dimensions of the US air governance system. Using a fixed effects model, we systematically quantify the efficacy of state policies on major pollutants in terms of intensity, political alignment, and regions.

In contrast to traditional manual methods, our text-mining approach, bolstered by indicator development and visualization, provides a holistic view of the US's air pollution control system. This approach fills the gap of previous studies by integrating multidimensional perspectives and quantifying the impacts of different factors. Moreover, it allows a comprehensive assessment of policy efficacy across different government tiers and pollutants. Drawing from these multidimensional analyses, this paper concluded with tailored policy suggestions, aiming to improve policy execution and accelerate the air quality improvement goals in the United States.

## Policy evolution based on environmental policy intensity

First, we calculate the average intensity scores of each document in our collection of the CAA and SIPs, discounting the effect of policy length to analyze the evolution of policy intensity. We also consider the effects of the multidimensional attributes on the evolution of policy intensity, as shown in the Appendices SA1 to SA4.

### Overall results

**Fig. 2.**
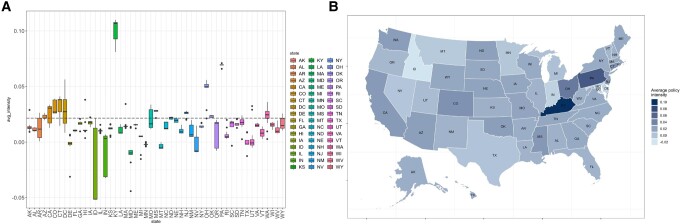
Comparing average intensity of SIPs across different states. (A) is the box plot of policy intensity across states, the horizontal dashed line in (A) represents the average intensity of the US CAA at the federal level. (B) is the average policy intensity across states.

Our data collection includes a range of policies from both federal and state governments, spanning decades. By aggregating all policies of each government, we can conduct a side-by-side comparison of the average intensity using a boxplot and a map, as shown in Fig. 2 (results of polarity and subjectivity are shown in Section SA1). This allows for a comparison between the federal government and each state, identified by their respective abbreviations.

From Fig. 2A, we can obtain several key insights. At the federal level, the CAA shows a moderately positive intensity, which suggests that the language in the CAA largely leans towards support, approval, and motivation. While states vary in policy intensity, the median intensity of most states aligns with the federal stance at a mildly positive level. While outliers exist, there is a general alignment of most states with the federal government. While there is a notable disparity in policy intensity range across states, as indicated by the box heights. States like Florida, Hawaii, and Illinois, show a relatively stable intensity across years, whereas states like California, Connecticut, and Oregon have undergone more fluctuations.

Comparing the average policy intensity of SIPs in each state (Fig. 2B), it can be found that Kentucky, Ohio, and Pennsylvania have significantly higher policy intensity than other states. Kentucky stands out with the highest positive intensity (0.1099) in 2016 and the highest average intensity of 0.1. In contrast, Idaho sinks to the most negative intensity (−0.05) from 2012 to 2018. In addition, Idaho's policy intensity has the most extensive range of (−0.05, 0.0214), positioning Idaho at the lowest intensity rank among all states.

### Policy evolution of the CAA

**Fig. 3.**
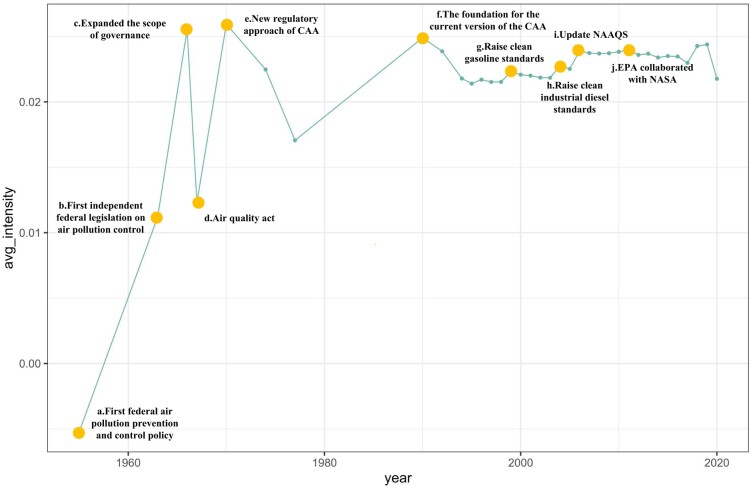
Policy evolution of the Clean Air Act.

The US federal law for air pollution control has come a long way since the first Air Pollution Control Act was passed in 1955 (32). With each policy document quantified by our NLP approach, Fig. 3 visualizes the evolving trajectory of the average intensity of these policies (the results of polarity and subjectivity are shown in Section SA2). Starting with a mildly negative intensity, the federal laws switched to the positive side with the promulgation of the original CAA in 1963. From then onwards, the intensity has generally demonstrated an upward tilt. During the 1960s and 1970s, federal laws went through several major milestones as indicated by the fluctuations in policy intensity. In particular, the CAA amendment of 1970, with the highest intensity score, established the fundamental structure for the US air pollution control system. After 1990, despite annual CAA updates, no major amendments to the CAA took place, and the intensity of federal laws remained largely consistent.

We spotlight several important policy adjustment points in Fig. 3 and analyze the evolution of the CAA in terms of the intensity scores (detailed descriptions in Appendix SA2). Clean air policies trace back as far as 1955 (point a), instigated by several severe air pollution crises, such as the photochemical smog pollution in Los Angeles in 1955, the Donora smog in 1948, and the Great Smog of Lonon in 1952, leading to the establishment of the Air Pollution Control Act. Initial policy adjustments (points b and c) led to a surge in the intensity of the clean air regulations. The significant intensification of the federal government's focus on air pollution control also prompted states like California and New York to enact emission standards for certain ambient air pollutants (32). However, the federal policy during this period advocated for regional regulation. This approach, along with voluntary state regulation, proved to be a setback because air pollution was not confined within regional boundaries. The lack of national-level emission standards resulted in local authorities failing in air pollution control. This also led to a decline in policy intensity after the 1967 policy reform (point d). In 1970, Congress transformed pollution control into a national responsibility, while supporting states through federal allocations to eventually establish viable state air control agencies in every state (32), demonstrating to the public that economic growth must be balanced with a cleaner environment. Based on its unprecedented success, the 1970 amendment was considered a watershed in the history of the CAA (point e). After two decades of experience and lessons learned from the implementation of the CAA, Congress amended the CAA again in 1990, establishing the legal structure of the current Act (point f). Since 1990, due to the well-developed policy system and the salient effect of air pollution control, the development of the CAA has stabilized (10). Although policy adjustments persisted (points g, h, i, j), their impact did not change the CAA's core, as indicated by the stable intensity scores.

### Political polarization in environmental policy

As climate change has been gaining attention in the United States, environmental policy has become a political arena between Republicans and Democrats (33). This polarization is part of the widening gap between the two parties (33). Although many scholars have attempted to dissect the partisan contrast within environmental policy, there is no consensus on the precise moment when this polarization intensified (10, 13, 34, 35).

**Fig. 4.**
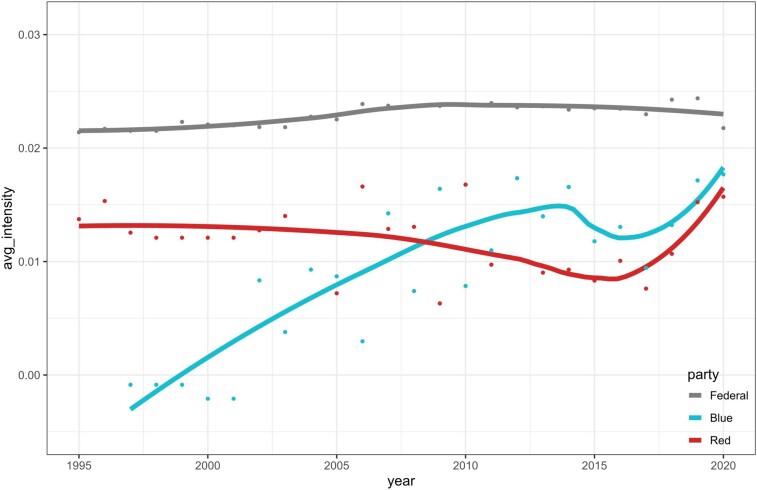
Political polarization in environmental policy.

Our exploration of this polarization delves into comparing air quality control policies of red (Republican-dominated) and blue (Democrat-dominated) states. Figure 4 shows the average policy intensity scores from 1995 to 2020 (the results of polarity and subjectivity are shown in Section SA3). During the past quarter-century, the federal policy intensity remained steady (∼0.023). In comparison, both red and blue states, showcased lower average intensity in their SIPs, indicating the federal government's primary stewardship in air pollution control. Notably, the turn of the 21st century saw a shift in both red and blue states’ focus on air quality control.

In the later 1990s, red states, with the SIP intensity averaging ∼0.015, outpaced the blue states (∼0.00). Before 2000, the CAA was designed to directly control air pollution from fixed or mobile sources, for instance, by installing emission control systems to protect public health, aligned with the Republicans’ ideas of reducing air pollution and risks (36). In contrast, post-2000, the CAA gravitated towards indirect emission reduction through the use of clean fuels and vehicles, like the policy on raising clean industrial diesel standards in 2004. This emphasis on clean and renewable energy was more in sync with the Democrats’ inclinations and divergent from the Republicans’ views (17, 19). Therefore, this phase, from the late 1990s to the early 21st century, marked a dip in the Republican policy intensity and a surge in the Democrats, narrowing the gap between the two.

Between 2005 and 2010, the average policy intensity of air pollution control between red and blue states was at a similar level. Post-2008 unveiled a significant divergence. Democratic-led states intensified their air pollution regulations, whereas their Republican counterparts seemed to take the opposite direction. Notably, while the intensity of red states plummeted to 0.009, blue states surged and reached a peak of policy intensity in the short term in 2014. This “widening gap” in air control policies resonates with the broader narrative of US political polarization (37). After 2014, despite the democrats maintaining a higher policy intensity, the two parties gradually synergized in their policy intensity and aligned closer to the federal standards. A possible explanation for this trend is that the federal government intensified lawsuits against states not complying with the CAA in recent years. According to official EPA data, administrative and civil judicial penalties assessed in areas of potential environmental justice concern increased from $14.87 million to $24.85 million between 2014 and 2020. Moreover, the overall number of defendants charged has remained at more than 100 since 2014 (38). The escalating costs of violations and frequent compliance checks prompted both parties to more actively seek policy coordination with the federal government, aiming to minimize the risk of potential punitive measures.

### Evolution of regional policy

**Fig. 5.**
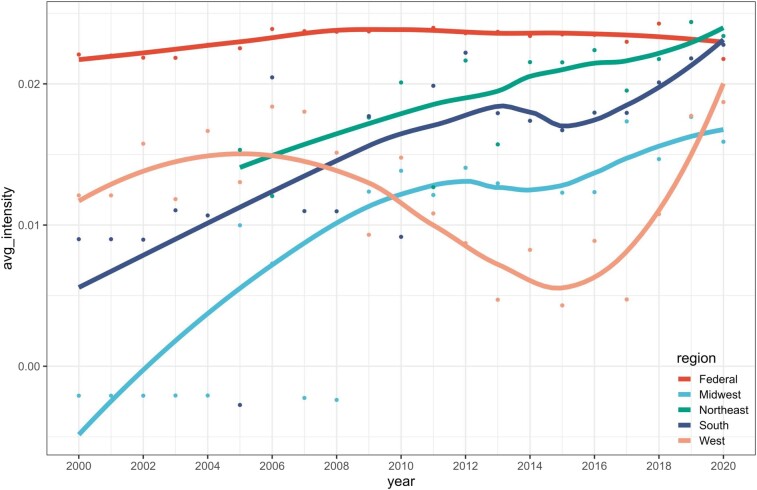
Policy evolution of four regions. Regional classification is based on the US Census Bureau, which is divided into the West, the South, the Midwest, and the Northeast.

The United States is commonly divided into regions—West, South, Midwest, and Northeast, each with a unique geography, development histories, and industrial focuses. These differences lead to regional policies in environmental governance. By aggregating the SIPs of different states from the four regions, we analyzed and compared the evolution of regional policies across the four regions. Figure 5 shows the average intensity of SIPs in the four regions since 2000 (the results of polarity and subjectivity are shown in Section SA4).

The Northeast prioritizes the prevention and control of industrial air pollution, evident by the highest policy intensity among the four regions. For its rigorous policies, the Northeast has achieved the lowest fixed pollution levels among all regions (as shown in Fig. SA5). The South, during its transition in its industrial structure, began to introduce strict air pollution control policies, leading to a consistent increase in policy intensity since the early 2000s. Over the past 10 years, the South's efforts to combat air pollution have aligned more with those of the Northeast. The West enforced the strictest pollution measures among the four regions between 2000 and 2006 and has fewer highly polluting industries that were reliant on fossil fuels. Post-2006, effective control of emissions from stationary facilities allowed the West to reduce its policy intensity while maintaining a level of around 0.01. Due to having fewer fixed sources of pollution compared to the South, the Midwest initially did not prioritize air pollution control as vigorously, leading to unsatisfactory environmental outcomes: by 1975, the EPA reported that 261 of the 394 coal-fired power plants in the United States were in compliance with SIP emission limitations or abatement schedules. Of the 133 plants not in compliance, 102 were located in the Midwest (39), which has significant delay and litigation around SO_x_ control plans (40). Over time, the Midwest has gradually intensified its policies and effectively decreased stationary sources emissions from 45.31 million in 2000 to 20.71 million in 2020 (41), a reduction of over 50%.

### Policy comparison between federal and state government

To dissect the relationship of air pollution policy between the federal and state governments, we investigate from both partisan and regional perspectives. Using the Maharaj distance ($d_{\mathrm{MAH}}$) and multidimensional scaling (MDS), Table 1, Panel A shows the pairwise distances of policy intensity among federal government, blue, and red states. There is a notable difference in policy intensity between red and blue states $\left(d_{\mathrm{MAH}}=1.37\right)$, suggesting greater political polarization between red and blue states. Comparing the disparity between red of blue states and the federal government, we found that since 1997, the heterogeneity between red states’ policy and federal policy is higher $\left(d_{\mathrm{MAH}}=1.74\right)$ and that the degree of homogeneity between blue states’ policy and the federal policy is the smallest among all the comparatives $\left(d_{\mathrm{MAH}}=0.15\right)$ (Fig. 4). This suggests that policy adjustments of blue states align more consistently with the federal government than the red states, especially at important moments.

**Table 1.** The differences and coordination of party, regional, and federal policies.

Index	Statistic results		*P*-value	
Panel A	Red States	Blue States	Red States	Blue States
Blue States	1.37		0.71	
Federal	1.74	0.15	0.63	0.69

Panel B	Midwest	Northeast	South	West	Midwest	Northeast	South	West
Northeast	0.32				0.96			
South	2.49	1.58			0.29	0.66		
West	1.98	0.85	0.94		0.58	0.84	0.82	
Federal	2.36	1.36	0.62	0.46	0.31	0.72	0.73	0.93

When *P*-value is not significant, the correlation sequences will be grouped together, and there is no significant difference in the dynamic structure of these sequences.

The comparison of policy intensity among the federal government and the four regions is shown in Table 1, Panel B, illustrating large disparities in their coordination with federal legislation. In particular, the West has a closer alignment with federal directives, evident in a $d_{\mathrm{MAH}}=0.46$. This is particularly pronounced during some important periods, such as 2006 and 2018. The Midwest shows the greatest divergence from the federal benchmarks, as indicated by a $d_{\mathrm{MAH}}=2.36$. This trend is due to opposing policy intensity shifts at specific points. For instance, the policy intensity in the Midwest showed a downward trend in 2005–2006 and 2017–2018, while the CAA of the federal government showed an upward trend.

The MDS results (Figs. SA6 and SA7) offer detailed insights. In terms of partisan factors, blue states, exemplified by California and Washington, have closely mirrored federal directives since 2010. In the last 5 years, more blue states such as New Jersey and New York, and even traditionally red states of Montana and Nebraska, have also trended toward federal policy alignments. Among the four regions, states in the West, such as Arizona and California, are in close sync with the federal government, with Texas (from the South) also in close affinity. In the first 15 years of the 21st century, states in the Northeast and Midwest had a similar distance from the federal government. In the last 5 years, states in the Northeast such as New Jersey and New York are closing their gaps with the federal government, showing signs of convergence.

Our results demonstrate the impact of both political party and regional factors on state-level policies for air pollution control. Nevertheless, pairwise tests indicate no statistically significant differences between parties or regions (Table 1). This finding indicates that, despite their political and regional disparities, the federal government is committed to ensuring a fundamental coherence between federal and state policies, through strong administrative measures, such as financial support (42).

## Environmental policy intensity and air pollutants

In this section, we delve into the relationship between the intensity of environmental policies and the levels of various air pollutants. Controlling for potential influences, we build a fixed effects model that attempts to examine how SIPs have reduced emissions of various pollutants over time, highlighting the roles of partisan and regional disparities.

### Overall policy effect

Table 2 presents how the SIP in each state has some effect on pollutant reduction. Summary statistics are shown in Table SB1, and a robustness test is shown in Section SB2. Overall, SIPs are associated with a significant decrease in the emission levels of all air pollutants such as CO, NO_x_, PM_10_, and PM_2.5_. Notably, the reduction in CO emissions is the most pronounced: an increase in policy intensity by one standard deviation (SD, here 63.57) corresponds to a decrease in CO emissions by 485 kilotons, i.e. 33% of the CO emissions’ SD. For particulate matter, the effect on PM_2.5_ emissions is greater than that on PM_10_: a one SD increase in policy intensity reduces PM_10_ and PM_2.5_ emissions by 109.98 kilotons and 46.45 kilotons, respectively, i.e. 35% and 45.7% of the SD of the respective pollutants. In contrast, SIPs have a less pronounced effect on NO_x_, while SO_2_ has not shown the same level of reduction. This distinction arises from specific measures implemented by the federal government at the end of the 20th and early 21st centuries to regulate these two pollutants. For instance, the NO_x_ Budget Program was introduced for NO_x_ by the federal government (43), while, a more diverse policy approach, including cap-and-trade and strong command-and-control regulations (44–46), was applied to SO_2_. These earlier interventions have led to substantial reductions in emissions, with NO_x_ declining by 71% and SO_2_ by over 94% since 1970 (47). Consequently, since the 21st century, with major achievements in managing SO_2_ and NO_x_ in the United States, there has been a discernible shift in policy focus toward other environmental challenges.

**Table 2.** Overall effect to different air pollutants.

	CO	NO_x_	PM_10_	PM_2.5_	SO_2_
Intensity	−7.63^***^	−0.27^**^	−1.73^***^	−0.73^***^	0.04
	(2.08)	(0.13)	(0.52)	(0.12)	(0.12)
CO NA	Y				
NO_x_ NA		Y			
PM_10_ NA			Y		
PM_2.5_ NA				Y	
SO_2_ NA					Y
CV	Y	Y	Y	Y	Y
State FE	Y	Y	Y	Y	Y
Party FE	Y	Y	Y	Y	Y
Year FE	Y	Y	Y	Y	Y
*N*	599	599	599	599	599

^*^Significant at 10%; ^**^significant at 5%; ^***^significant at 1%.

CV, control variables.

An alternative approach to assess the magnitude of policy intensity involves comparing their explanatory power against other variables in the fixed-effect regression model (48) by excluding the effects of state, party, and year. Via analyzing the data from Table 2 and performing a standard variance decomposition, we found that policy intensity and other covariates collectively account for 5.15% of the variance in CO emissions. Within this 5.15%, 18.43–48.7% is explained by policy intensity. Further comparisons of the explanatory power of policy intensity on PM_10_ and PM_2.5_ emissions reveals that policy intensity explains 4.03% of the total variance in PM_2.5_ emissions, with 43.07–47.12% of this variance resulting from policy intensity. In contrast, policy intensity accounts for 3.33% of the variance in PM_10_ emissions, with 21.1–24.53% attributed to policy intensity. The influence of policy intensity on NO_x_ and SO_2_ emissions is less obvious.

### Policy effects influenced by different policy tools

In understanding how policies affect environmental outcomes, the intensity of the policy languages serves as an informative metric. As shown in examples in Table SC2, policy texts utilize expressions of both positive and negative intensity. While positive-intensity phrases aim to foster and incentivize behaviors, negative-intensity phrases imply punishments or prohibitions. Here, we analyzed the impact of total policy intensity on various air pollutants. Furthermore, we also dissect policy intensity into its positive and negative measures, offering a more granular view of how these two sides individually affect emission reductions. The results are summarized in Table 3 (the robustness test is shown in Section SB3).

**Table 3.** Policy effects of different directional intensity.

	CO		NO_x_		PM_10_		PM_2.5_		SO_2_	
	*P*	*N*	*P*	*N*	*P*	*N*	*P*	*N*	*P*	*N*
Intensity	−6.50^***^	−3.84	−0.26^**^	−0.67	−1.51^***^	−0.88	−0.61^***^	−0.27	0.08	0.82^**^
	(1.77)	(4.53)	(0.12)	(0.56)	(0.51)	(1.62)	(0.11)	(0.30)	(0.09)	(0.35)
CO NA	Y	Y								
NO_x_ NA			Y	Y						
PM_10_ NA					Y	Y				
PM_2.5_ NA							Y	Y		
SO_2_ NA									Y	Y
CV	Y	Y	Y	Y	Y	Y	Y	Y	Y	Y
State FE	Y	Y	Y	Y	Y	Y	Y	Y	Y	Y
Party FE	Y	Y	Y	Y	Y	Y	Y	Y	Y	Y
Year FE	Y	Y	Y	Y	Y	Y	Y	Y	Y	Y
*N*	599	599	599	599	599	599	599	599	599	599

^*^Significant at 10%; ^**^significant at 5%; ^***^significant at 1%.

CV, control variables; P, positive; N, negative.

The results indicate that policies with positive intensity have a primary influence in shaping outcomes. For instance, an increase in positive intensity by 1 SD (here 84.45) would reduce CO emissions by 548.9 kilotons, i.e. 37% of the CO emissions’ SD. Similarly, policies with positive intensity will also decrease the emissions of PM_10_ and PM_2.5_ by 127.5 and 51.51 kilotons, respectively, i.e. 40 and 50% of the SD of the corresponding pollutants. Although policies with negative intensity can help reduce pollutant emissions to some extent, their potency appears to be limited for most pollutants. There is a positive correlation between negative-intensity policy and SO_2_ emissions, which may be due to the fact that the sum of negative intensity is much smaller than that of positive intensity, as shown in Fig. SA8. This suggests that US states have preferred more lenient and flexible policy expressions since the 21st century. Furthermore, the magnitude of evolution in negative-intensity policy has been relatively small, especially post-2010, where the intensity remained near 10, suggesting that negative-intensity policies have not functioned as a major policy instrument in recent years. This is consistent with the trend that SO_2_ emissions have remained relatively stable since the 21st century.

Comparing the effects of overall intensity and positive intensity reveals that policies with positive intensity are more effective in promoting the reduction of emissions across all pollutant types. However, policies oriented to negative intensity remain crucial, as demonstrated by the federal government's increased litigation efforts in recent years against states failing to comply with the CAA (38). Positive strategies, such as fostering innovation and providing economic incentives, when combined with negative policies, such as establishing rigorous standards and imposing penalties, can ensure that regulated entities adhere to the required limits and that the policy's fundamental goals are attained.

### Policy effects influenced by political polarization

This section delves into the impact of partisan differences on SIPs, highlighting a significant disparity between red states and blue states in terms of pollution control effectiveness (Table 4), confirming the assertion that “conservatives tend to be less concerned about the harmful effects of air pollution” (49). Overall, the SIPs of blue states have shown more significant effects in recent years, particularly reducing almost all harmful pollutants, except for SO_2_. This improvement in regional air quality represents a further development beyond conclusions drawn in prior research (2). The reduction in CO emissions was particularly significant. An increase in policy intensity by 1 SD (here 103.6) resulted in a reduction in CO emissions by 1372.7 kilotons, i.e. 93% of the CO emissions’ SD. This represents a notable difference compared to reductions for other pollutants. The lesser emphasis on SO_2_ emission control in blue states is attributed to the rigorous federal and state government management of sulfide in the late 20th century. Given these previous efforts, blue states did not allocate significant resources to sulfide management in recent years, leading to diminishing policy effects.

**Table 4.** Heterogeneity effect of different parties.

	CO		NO_X_		PM_10_		PM_2.5_		SO_2_	
	Blue	Red	Blue	Red	Blue	Red	Blue	Red	Blue	Red
Intensity	−13.25^***^	4.14	−0.28^***^	1.60^**^	−1.94^***^	4.64	−1.15^***^	0.41	0.04	1.34
	(3.06)	(8.08)	(0.05)	(0.56)	(0.23)	(2.60)	(0.23)	(0.87)	(0.12)	(0.87)
CO NA	Y	Y								
NO_X_ NA			Y	Y						
PM_10_ NA					Y	Y				
PM_2.5_ NA							Y	Y		
SO_2_ NA									Y	Y
CV	Y	Y	Y	Y	Y	Y	Y	Y	Y	Y
State FE	Y	Y	Y	Y	Y	Y	Y	Y	Y	Y
Party FE	Y	Y	Y	Y	Y	Y	Y	Y	Y	Y
Year FE	Y	Y	Y	Y	Y	Y	Y	Y	Y	Y
*N*	192	153	192	153	192	153	192	153	192	153

^*^Significant at 10%; ^**^significant at 5%; ^***^significant at 1%. CV, control variables.

Red states did not show significant mitigation efforts on emissions of all air pollutants. This ineffectiveness in air pollution governance is related to their partisan affiliation and the negative effects of partisan polarization in recent years. This disparity in policy intensity and governance efficacy reflects the contrasting priorities Democrats and Republicans assign to environmental issues. A Pew Research Center survey revealed that only 10% of Republicans and Republican-leaning independents consider the climate issue a personal top priority, compared to 49% of Democrats and Democratic leaners (50). The political polarization toward climate change is also reflected in attitudes toward different types of climate policy. Conservatives (or Republicans) typically express less concern about the environmental impacts of energy development such as water and air pollution (49, 51). Their support for policies is often driven by economic cost/benefit considerations (17). For instance, policies that favor subsidized coal-fired power plants are more likely to get support from conservatives and opposition from liberals (52). Furthermore, for economic and interest reasons, the Republican majority has been associated with reducing the new expenditures in air pollution abatement and the tendency of adopting the most effective NO_x_ abatement technology in nonattainment areas (2).

### Policy effects influenced by regional factors

This section focuses on comparing the effects of SIPs on air pollutants across regions (results are shown in Table 5). The results reveal that states in the West demonstrated optimal air quality control outcomes, showing a significant inhibitory effect on all major air pollutants. Particularly notable was the abatement of CO: an increase in 1 SD (here 115.5) would reduce CO emissions by 1492.26 kilotons, i.e. 78.9% of the CO emissions’ SD, making the West the leading region among all. From 1997 to 2020, the states in the West focused on the control of particulate matter (PM), leading to effective management of PM_10_ and PM_2.5_., while the control of traditional toxic gases (SO_2_ and NO_x_) was less effective.

**Table 5.** Heterogeneity effect of different regions.

	Midwest	Northeast	South	West
CO	−0.30	−3.13^**^	0.22	−12.92^***^
	(1.29)	(1.31)	(1.48)	(2.71)
NO_x_	−0.26	−0.53	−0.03	−0.31^***^
	(0.25)	(0.43)	(0.20)	(0.08)
PM_10_	−1.47^*^	0.12	−0.07	−2.29^***^
	(0.73)	(0.40)	(0.48)	(0.37)
PM_2.5_	−0.05	−0.03	0.04	−1.14^***^
	(0.12)	(0.08)	(0.11)	(0.23)
SO_2_	−0.06	−0.54	0.02	−0.07^***^
	(0.43)	(0.51)	(0.17)	(0.02)

^*^Significant at 10%; ^**^significant at 5%; ^***^significant at 1%.

Compared to the West, the Northeast displayed limited policy effects. Despite being less adept at CO reduction, an increase in policy intensity by 1 SD would reduce CO emissions by 8% in their SD. As shown in Fig. SA5, the Northeast has the lowest emissions from stationary sources among all four regions. Therefore, the region has refrained from frequent policy updates since the turn of 21st century, leading to a diminishing impact of these policies. In comparison, the Midwest's and the South's policies exhibited limited effectiveness. The policy effect of PM_10_ in the Midwest was inferior to the West, an increase in policy intensity by one SD would reduce PM_10_ emissions by only 8.8% of their SD. For other pollutants, the policy's effectiveness was not pronounced. Although all pollutants had a downward trend in the Midwest, there is a need for more stringent policy governance for air pollution control.

Likewise, the South's policies exhibited limited effectiveness in curbing air pollutant emissions. Their policy intensity seems to be “decoupled” from the emissions for most pollutants, except for weak effects on NO_x_ and PM_10_. Specifically, Alabama, Mississippi, and Oklahoma maintained almost constant policy intensity over decades, suggesting stagnant SIP updates and diminishing regulatory ability over time. Conversely, states like Florida, Georgia, and Kentucky showed a downward trend in policy intensity. Some states like Delaware, Maryland, South Carolina, and Texas even exhibited negative policy intensity in certain years. The South had the lowest overall policy intensity among all regions, which did not benefit their air quality, in terms of both fixed pollution sources and corresponding emissions.

## Discussion

This article introduces a novel NLP method to construct a policy intensity index capturing the prolonged evolution in US air governance policy at various levels. We employed Maharaj Distance and MDS to analyze the alignment between state and federal policies in terms of partisan and regional dimensions. We then used the fixed effects model to examine the mitigation effects of policy on air pollutants and to discern the influence of intensity, political polarization, and regional characteristics on policy efficacy.

At the federal level, the 1970 and 1990 amendments stood out in intensity due to their monumental importance in the long history of clean air policy. Since the late 1990s, environmental laws have gradually become a contested domain between Republicans and Democrats. Our results show a pronounced 1.37 difference in average intensity between red and blue states. Blue states, showing a closer alignment with the federal government with a mere difference of 0.15, contrast starkly with the red states’ 1.74. From the regional aspect, the Northeast has the highest average policy intensity, while the West exhibits the closest alignment with the federal government, evidenced by the smallest Maharaj Distance of 0.46.

Empirical results show that policy enactment significantly mitigated emissions of all pollutants except SO_2_, which provides preliminary support for the continued use of policy tools to reduce pollutant emissions in the future. The most significant effect was observed for CO emissions, where an increase in policy intensity by 1 SD led to a reduction of 485 kilotons, i.e. 33% of the CO emissions’ SD. While positive intensity plays a critical role in pollutant reduction and negative intensity ensures basic policy efficacy, employing a combination of policy instruments offer greater robustness. Both partisan polarization and regional disparities have notable impacts on policy outcomes. From the partisan standpoint, while blue states exhibited effective emission suppression for most pollutants, red states lagged. Regional comparisons show that the West led in overall effectiveness, followed by the Northeast, while the Midwest and South showed varying degrees of stagnation or even regression, culminating in subpar outcomes.

A variety of policy options can bridge the gaps in policy effects across political affiliations and regions. The recent years have seen a gradual convergence of policy intensity across states between red and blue states, mainly due to the increasing costs imposed by the federal government for noncompliance by state governments. This has prompted a synergistic and passive behavior from state governments. While there is a broad consensus among the American public on the urgency of combating climate change (53), divisions persist on specific environmental issues (54). Therefore, employing a diverse set of policy tools to bridge the gap between the two parties can help sustain the effectiveness of environmental policies. For example, bipartisan support can be achieved through policies that offer incentives and minimize impact on the economy and job market, such as tax credits and advancing new energy vehicle technologies. Furthermore, amplifying the significance of air governance can sway younger demographics to endorse air governance policies.

Regionally, while the West and Northeast have achieved remarkable success in air governance, challenges persist. The West requires a sustained investment of resources to tackle air pollution from mobile sources due to its highest automobile registrations by the end of 2021 (55). For the Northeast, the focus of future policymaking needs to be pivoted to managing NO_x_ and particulate matter. For the Midwest and South, with historically weak policy intensity, federal intervention may be necessary by raising pollution compliance in certain areas and intensifying spot checks in areas to ensure SIP adherence. In addition, most states in the Northeast, Midwest, and South are bound by the Good Neighbor Plan enacted by the EPA. This plan ensures that the regulatory impact extends beyond local regulations, affecting the air quality in the downwind states as well (56). Therefore, air pollution control in these regions will not only produce local environmental benefits, but it is projected to generate approximately $4.3–15 billion for the US economy in the future (57).

Our study investigates a multifaceted policy dataset, offering critical practical significance to both researchers and policymakers. Future research can utilize our proposed methodology to examine extant theories that previously lacked adequate long-term measures and build research frameworks to scientifically assess the causal effects of policies in regard to factors like community, economic investment, and governing bodies. Our measurement tools can reveal details and patterns hidden in the policy text. Such insights, extended to additional policy, language, and contexts, not only render historical policy trajectories but also offer a blueprint for future policy design and enactment.

## Materials and methods

### Data collection and processing

We collected policy texts from websites such as EPA, JUSTIA, Cornell Law School, and the state legislature websites. After comprehensively reviewing the policies of the federal and state governments, we manually gathered the relevant policy texts. For each policy document, we recorded its associated metadata (e.g. government level and year) and ensured data integrity by reviewing its content. We applied a consistent naming convention to all data files and built a collection of policy documents. Specifically, the collection contains 36 federal-level policy documents from 1955 to 2020, and 607 state-level SIP documents covering all fifty states and the District of Columbia from 1973 to 2020 (basic information on SIPs is in Table SC1). A detailed breakdown and analysis are provided in Section SC1.

### Measuring environmental policy intensity

Policy documents serve as the “vehicle” of policies, enabling researchers to study the primary policy content, the policymaking process, and the policy instruments (58). Archiving policy documents over an extended period can offer insights into policy evolution. This is an exploratory process where the government crafts rules according to the social, economic, and technological demands of different eras (10, 59). However, traditional qualitative analysis methods are not equipped for handling vast policy documents or comparing the shifts in policy intensity over time. In contrast, a quantitative method by converting policy texts into numerical data, can address these challenges.

We employ NLP methods to reveal the partisan and regional nuances embedded in the CAA by constructing a policy intensity index. In recent years, text analysis has proven instrumental for the quantitative analysis of various text documents (60–62). The NLP approach adopted here blends a vocabulary-based linguistic approach with machine learning (63). It allows analyzing extensive texts and converting human language into a structured, machine-readable format (64–66). For our purpose, we used an NLP toolkit (67) to analyze the policy documents of the CAA and SIPs. Given a document (or a sentence) as input, this toolkit can produce two scores: polarity and subjectivity.

We define “intensity” as an index that quantifies policy instruments based on the stringency and importance of policy (68–70). While the literature introduces several related constructs, such as “importance,” “significance,” and “stringency” (71, 72). We contend that, in the context of policy texts, polarity reflects the government's usage of positive or negative language to express incentivized or prohibited behaviors; and subjectivity indicates the government's commitment and urgency in achieving environmental goals. Drawing from prior research on policy intensity (68, 73), we define the following index:


(1) $$\begin{array}{c} \mathrm{Intensity}=\mathrm{Polarity}\;\times \;\mathrm{Subjectivity}.\; \end{array}$$

Section SC2 provides more details, including the interpretation, value ranges, and computation result of polarity, subjectivity, and intensity, using five exemplar sentences from the 1990 CAA amendment and average policy intensity results in each state. These examples demonstrated the NLP toolkit's efficacy in capturing and differentiating the orientation and vigor of policy instruments established in government policies. A more detailed analysis process is depicted in Table SC3.

### Comparing differences and coordination between federal and state governments

To measure the dissimilarity between two time series, we used the Maharaj distance. The Maharaj distance (74, 75) utilizes hypothesis testing to determine whether two autoregressive-moving-average time series, $X_{T}$ and $Y_{T}$, are generated by two significantly distinct processes. By constructing indicators, $\mathrm{dissimilarit}y_{\mathrm{MAH}}(d_{\mathrm{MAH}})$ and its *P*-value ($d_{\mathrm{MAH},p}(X_{T},Y_{T})$), we can measure the level of disparity between $\;X_{T}$ and $Y_{T}$. The formula and the interpretation of the corresponding parameters (Appendix, Table SC4) are shown below:


(2) $$\begin{array}{c} d_{\mathrm{MAH}}(X_{T},Y_{T})=\sqrt{T}{\left({\hat{\Pi }}_{X_{T}}^{\prime }-{\hat{\Pi }}_{Y_{T}}^{\prime }\right)}^{T}{\hat{V}}^{-1}({\hat{\Pi }}_{X_{T}}^{\prime }-{\hat{\Pi }}_{Y_{T}}^{\prime }) \end{array}$$

(3) $$\begin{array}{c} d_{\mathrm{MAH},p}(X_{T},Y_{T})=P(\;\chi_{k}^{2}>d_{\mathrm{MAH}}(X_{T},Y_{T}))\; \end{array}$$

Both test statistics, $d_{\mathrm{MAN}}$ and $d_{\mathrm{MAH},p}$, satisfy the properties of non-negativity and symmetry. As such, one of the statistics can serve to measure the difference between $X_{T}$ and $Y_{T}$. With $d_{\mathrm{MAN}}$, a greater value denotes a more pronounced difference between the two time series. By setting a significance level *α*, we can use $d_{\mathrm{MAH},p}$ as an index to measure the difference. If $d_{\mathrm{MAH},p}>\alpha$, the correlation sequences will be grouped together, indicating no significant disparity in the dynamic structure of the sequences.

### Multidimensional scaling

MDS is a multivariate statistical analysis method that uses correlation ranking for information visualization and dimensionality reduction. It enables the visual representation of similarities (76, 77) and has been frequently used in previous studies (78–81). In this article, we analyze the texts of CAA and SIPs, utilizing term frequencies as the multidimensional features of each policy document. The MDS technique then reduces the similarity relationship among documents from a multidimensional space to a 2D space.

By assigning features of partisan and regional attributes to different states, the distances visualized in the 2D space between the federal and the state levels can further complement the earlier results on the differentiation and coordination between the two tiers of government. the specific analysis steps are as follows: (i) constructing an initial text corpus through preprocessing the policy texts; (ii) formulating the document-term matrix and removing terms with a frequency below 0.5% from the matrix; (iii) normalizing the term frequency matrix and calculating the Euclidean distance between multidimensional vectors in the normalized matrix; and (iv) MDS is applied to the distance matrix for dimensionality reduction and visually show the distance between different texts in the 2D space.

### Evaluating policy effect on pollutants

A two-way fixed-effect model is constructed for exploring the potential effects of policy intensity on air pollutant emissions. Emissions of major air pollutants (CO, NO_x_, PM_10_, PM_2.5_, SO_2_), as delineated in the NAAQS (82, 83), serve as the dependent variables, while policy intensity stands as the independent variable. To counteract biases stemming from unobservable variables at both the time and regional level, we control for time and region fixed effects. The baseline empirical model is shown in equation (4):


(4) $$\begin{array}{c} \mathrm{Pollut}e_{ijt}=\beta \mathrm{Intensit}y_{it}+\delta_{i}+\sigma_{t}+\varepsilon_{it} \end{array},$$

where $\mathrm{Pollut}e_{ijt}$ denotes the emissions of pollutant *j* in state *i* in year *t*, $\mathrm{Intensit}y_{it}$ denotes the annual policy intensity of state *i* in year *t*, $\delta_{i}$ denotes the state's fixed effect and $\sigma_{t}$ denotes the year's fixed effect.

The NAAQS establishes the minimum and uniform ambient air quality levels that the CAA mandates the EPA to set for the six pollutants in the United States. When a county's air quality falls below the NAAQS standards, it is designated as a “nonattainment” area, more stringent EPA regulations until local emissions decrease and the air quality meets or exceeds the thresholds. Furthermore, when a county is labeled a “nonattainment” area, the state must re-evaluate its SIP to ensure compliance by regulating all major sources of pollution (84). Therefore, the number and duration of counties designated as “nonattainment” areas within a state can profoundly influence the policy intensity, and subsequently local air pollution control. Given the provisions of the NAAQS and the available data, this study considers the major pollutants, excluding Pb and O_3_, as the dependent variables while controlling for the count of “nonattainment” areas.

In addition, this article accounts for a state's basic conditions, industrial composition, and coal consumption as control variables (84) to negate potential effects from factors other than policy intensity on air pollutants. The definition and measurements of these variables can be found in Table SC5. Augmenting the baseline empirical model leads to the policy assessment model as follows:


(5) $$\begin{array}{c} \mathrm{Pollut}e_{ijt}=\beta \mathrm{Intensit}y_{it}+\gamma X_{it}+\delta_{i}+\sigma_{t}+\varepsilon_{it} \end{array},$$

where $X_{it}$ denotes the control variables mentioned above; the other variables are the same as those in equation (4).

## Supplementary Material

pgae199_Supplementary_Data

## Data Availability

The policy text data used in this study is publicly available and can be obtained from the EPA, JUSTIA, Cornell Law School, and the United States House of Representatives. The code and packages used for analysis are executed in R (4.3.1) and Python (3.10.13) programming languages. All code and data have been uploaded to the public data repository *figshare* (85).
