# Supplementary material for: Quantifying US air pollution policy: How political and regional factors influence pollutant mitigation
Source: PNAS Nexus. 2024 May 17;3(5):pgae199. doi: 10.1093/pnasnexus/pgae199 (PMC11138116; doi:10.1093/pnasnexus/pgae199)
Supplement: pgae199_Supplementary_Data [file pgae199_supplementary_data.docx]

**Supporting Information**

**Quantifying U.S. air pollution policy: How political and regional factors influence pollutant mitigation**

Guoxing Zhang, Zhanglei Chen, Jiexun Li, Bin Su, Yang Gao, Lean Yu

**Contents**

[A. Policy evolution based on environmental policy intensity 2](#_Toc160033263)

[A1. Overall results 2](#_Toc160033264)

[A2. Policy evolution of the Clean Air Act 3](#_Toc160033266)

[A3. Political polarization in environmental policy 5](#_Toc160033268)

[A4. Evolution of region policy 6](#_Toc160033270)

[A5. Fixed pollution of four regions 8](#_Toc160033272)

[A6. MDS results for different parties, different regions vs federal government 10](#_Toc160033275)

[A7. Policy intensity comparison 12](#_Toc160033278)

[B. Data description and testing 13](#_Toc160033280)

[B1. Descriptive data 13](#_Toc160033281)

[B2. Robust test of overall policy effect 14](#_Toc160033283)

[B3. Robust test of policy effect of positive and negative intensity 18](#_Toc160033287)

[C. Materials and Methods 26](#_Toc160033294)

[C1. Policy basic features 26](#_Toc160033295)

[C2. Text preprocessing of policy documents 29](#_Toc160033298)

[SI References 37](#_Toc160033300)

# A. Policy evolution based on environmental policy intensity

**A1. Overall results**

**Fig. A1.** Comparing average polarity and subjectivity of clean air policies across different governments.

**A2. Policy evolution of the Clean Air Act**

Regarding polarity (section A2, Figure A2(A)), we find that the polarity of federal laws is mostly positive and that the 1970 amendment to the CAA was the policy document with the most positive polarity. The polarity of the 1990 amendment was slightly lower than that of the 1970 one. In general, federal laws place more stress on the support and promotion of air governance. Section A2, Figure A2(B) reveals high subjectivity (~0.3) in earlier policy, indicating the federal government’s commitment to addressing air pollution. In 1995, the EPA started to collaborate with other departments, such as the National Oceanic and Atmospheric Administration (NOAA), to use remote sensing data for real-time and accurate monitoring of the atmospheric status (1). These new technologies and collaborations aid the EPA in air pollution control decisions.

**Fig. A****2.** The evolution of policy polarity and subjectivity of the Clean Air Act.

**Policy evolution of the Clean Air Act.**

**(1) Before 1970-Initial establishment of the CAA.** Before 1955, there was no national air pollution control policy in the U.S. However, economic development at the expense of the environment endangered the health and well-being of the Americans. For example, in 1948, the Donora smog incident, which lasted more than 5 days, resulted in 20 deaths and 6,000 illnesses. The Great Smog incident in London in 1952 also sounded the alarm clock for American air pollution control. In 1955, Los Angeles experienced severe photochemical smog pollution (2), which led to the passage of the Air Pollution Control Act (point a). This was the first federal policy to address air pollution and control. In 1963, the federal government passed the CAA (point b), which was the first law to promote public health and welfare. This paved the way for future amendments to the CAA and heightened its intensity. Three years later (point c), the federal government expanded the scope of governance by setting automobile exhaust standards and establishing air quality control areas among other measures. In 1967 (point d), the federal government attempted to coordinate actions between local governments and industries to address air pollution problems on a regional basis. However, this strategy proved impractical and yielded inadequate pollution control results. Consequently, the policy intensity decreased abruptly.

**(2) The 1970 amendment of the CAA.** On April 22, 1970, the first Earth Day was held, and approximately 20 million people participated in the largest social event. Congress amended the CAA in 1970 (point e) in response to public pressure generated by the Earth Day campaign and years of failure to control air pollution. This amendment established national air quality standards and statutory deadlines for the first time and launched four monitoring programs for emissions from fixed sources: the NAAQS, SIPs, the New Source Pollution Standards (NSPS), and the National Emission Standards for Hazardous Air Pollutants (NESHAP). In addition, Congress crated the Environmental Protection Agency (EPA) to specifically address environmental issues, thereby expanding the federal government’s authority over air pollution control and establishing a statutory deadline for emission standards for each pollutant in order to strengthen CAA requirements. Through the 1970 amendment, Congress transformed pollution control into a national responsibility, demonstrating to the public that economic growth must be balanced by a cleaner environment. Based on its unprecedented success, the 1970 amendment was considered a turning point in the history of the CAA. Additionally, in 1970, the CAA’s policy intensity reached its peak.

**(3) The 1990 amendment of the CAA.** Congress amended the CAA again in 1990, establishing the current Act’s legal framework (point f). The new CAA was first incorporated as a subchapter into the United States Code. The new law included stricter vehicle emission standards, the promotion of low-sulfur fuels and alternative fuels to solve the air pollution problem caused by traditional pollutants, and the elimination of chlorofluorocarbon (CFC) compounds to reduce the destruction of the ozone layer. Congress paid more attention to the joint control of air pollution through cooperation and consultation with all levels of government and non-governmental organizations. Furthermore, the 1990 amendment was more adaptable and result-focused. Rather than adhering to a fixed model or specific requirements, market factors were considered when designing policies, providing great opportunities for governments and enterprises at all levels. Various instruments were enacted, such as providing certain material rewards or credit support to companies that can take prompt action to reduce toxic emissions or pollutant emissions below legal standards, providing clean vehicle manufacturers and vehicles that require clean fuel with tradable emission rights, and reconfiguring fuel performance indicators to assist industrial enterprises in attaining emission reduction goals in the most cost-effective manner (3). The 1990 amendment laid the groundwork for the current version of the CAA and established a precedent for the formulation of economically effective environmental policies worldwide. Since then, market factors have become crucial considerations for other governments when formulating environmental protection policies.

**(4) 1990 to present- Gradual stabilization of CAA.** Since 1990, the development of the CAA has been stabilized due to the well-developed policy system and the prominent effect of air pollution control (3). Here, the evolution of policy was primarily reflected in the clarification and improvement of standards and in minor modifications to their scope. For example, regarding clean fuel standards, Congress raised clean gasoline standards and clean industrial diesel standards in 1999 (g) and 2004 (h), respectively. In 2006 (i), the EPA revised the NAAQS to establish stricter standards for fine particulate pollution. In 2011 (j), the EPA collaborated in DISCOVER-AQ with NASA and others to better understand how to measure and forecast air quality globally via satellite data from space (4). However, these modifications did not alter the overall CAA structure, as evidenced by the stable intensity scores.

**A3. Political polarization in environmental policy**

On the basis of the historical presidential elections results since 1996, we identify states that have consistently chosen Democrats as blue states and states that have consistently chosen Republicans as red states. In summary, we selected 14 red states (Alaska, Alabama, Idaho, Kansas, Mississippi, Montana， North Dakota, Nebraska, Oklahoma, South Carolina, South Dakota, Texas, Utah and Wyoming) and 16 blue states (California, Connecticut, District of Columbia, Delaware, Hawaii, Illinois, Massachusetts, Maryland, Maine, Minnesota, New Jersey, New York, Oregon, Rhode Island, Vermont and Washington) for the analysis.

**Fig. A****3.** The evolution of policy polarity and subjectivity of deep red states and deep blue states.

Based on the analysis of policy polarity and subjectivity (section A3 Figure A3 (A) and Figure A3 (B)), since 1990, the policy orientation of the Democratic Party has shifted from negative to positive, and air pollution control has shifted from negative means such as command and control to positive means such as advocacy and promotion. Since 1990, the overall stance of the Republican Party towards air pollution control has been consistently positive. Between 2000-2015, the polarity of Republican policy tended to remain relatively stable, whereas Democratic policy was still undergoing a period of dynamic adjustment, and polarity continued to increase. But after 2016, both Democrats and Republicans agreed to raise the policy polarity by using more encouraging policy tools to manage air pollution. Although the subjectivity of the Democratic Party’s policy showed an overall upward trend, its overall subjectivity score was less than 0.2, and the change range of policy subjectivity decreased after 2010 and gradually decreased at the beginning of 2018, indicating a shift toward objective description. In contrast, the subjectivity score of the Republican policy was greater than 0.2 over the course of the year. In 1993, the subjectivity of Republican policy reached 0.27. This suggests that Republicans are more likely to use more subjective statements in environmental policy formulation. The policy polarity outcome indicates that Republicans' utilization of positive policy statements has marginally declined in recent decades, whereas policy subjectivity has consistently remained above 0.2. This suggests that Republicans have not placed significant emphasis on the implementation of supportive and incentive policies as instrumental tools in environmental governance in the past. Instead, they have incorporated subjective descriptions of policies lacking instrumental attributes.

**A4. Evolution of region policy**

Comparing the policy polarity and subjectivity of the four regions (section A4, Figure A4), we find that due to its unique position as a political, economic, and cultural hub, the Northeast displayed a more positive and open attitude towards air pollution control in general, and the air pollution control policies of the Northeast also exhibited more scientific and objective characteristics. Before 2010, the South experienced significant fluctuations. In 2005, the policy polarity abruptly shifted from positive to negative, and the subjectivity level rose from 0.175 to 0.25, which is related to the subjectively negative regulatory policies introduced by Maryland and Minnesota that year. After 2010, policy polarity, subjectivity, and intensity levels in the South gradually flattened.

**Fig. A****4.** The evolution of policy polarity and subjectivity of the four regions.

In general, the policy indicators for the Midwest have exhibited an upward trend; however, there were two years in which they underwent abrupt changes: a sudden increase in 2005 and a sudden decrease in 2007. Significant changes in the air control policies of Illinois and Missouri in 2005 led to the sudden increase in 2005 policy indicators. Illinois’s SIPs in 2005 had the highest polarity, subjectivity and policy intensity over the years. Missouri’s SIPs in 2005 were also a relatively positive and subjective policy. In 2007, policy indicators in the Midwest declined due to the fact that other states didn’t revise their SIPs in that year, whereas Minnesota continued to implement pa relatively punitive and objective policy, thereby altering the polarity and subjectivity of policy in the Midwest as a whole that year.

The West has a smaller change range of policy polarity and subjectivity than the other three regions. In the West, there is a slight downward trend in policy polarity, whereas policy subjectivity is always adjusted to approximately 0.225. Under the comprehensive effect, policy intensity in the West tends to decline slightly.

**Evolution of regional policy.**

The Northeast is the most prosperous region and is home to the nation’s capital, Washington, D.C.. The region relied heavily on industry for its economy before the 1940s. Due to its strategic economic, political and cultural location, the Northeast has paid more attention to the prevention and control of industrial air pollution and air quality than the other three regions and has the highest policy intensity among the four. In pursuit of lower production costs, the majority of traditional and heavy industries were transferred to other states or countries during the second half of the 20^th^ century, and the region developed low-carbon industries such as microelectronics, computers and biotechnology. Consequently, the region had the lowest levels of fixed pollution among the four regions (section A5, Figure A5). Emerging industries with high added value not only provide financial support for the pollution prevention plans of state governments but also provide a material foundation for achieving air control goals through high technology.

The South possesses an abundance of natural gas and oil. The region’s primary economic sectors are oil and gas extraction, quarrying and mining, all of which are extremely polluting (section A5, Table A1). Employment and wage growth have been stimulated by the region's vast oil and gas reserves, which have also spawned the development of industries with substantial fixed pollution emissions (section A5, Figure A5). Since the turn of the 21^st^ century, the South has consistently increased the intensity of its air pollution control policies in an effort to realign its industrial structure. Over the past decade, policy intensity has increased significantly, bringing the South’s air pollution mitigation efforts in line with those of the Northeast. Notwithstanding economic and political disparities, Southern governments maintain their resolute commitment to addressing this issue and guaranteeing a more salubrious environment for all.

Compared to the other three regions, the West possessed a greater abundance of renewable resources, as evidenced by the Alta Wind Energy Center (AWEC) in California and the Grand Coulee Dam in Washington. Therefore, there were fewer highly polluting industries reliant on fossil fuels in the West. From 2000 to 2006, the West enforced the strictest pollution treatment among the four regions. After 2006, the West gradually decreased its air policy intensity and maintained a level of approximately 0.01, owing to the efficient regulation of emissions from its stationary facility.

The Midwest functioned as a pivotal hub for manufacturing, not only within the U.S., but also on an international scale. Consequently, numerous factories discharged substantial levels of pollutants (section A5, Table A1) from stationary sources (section A5, Figure A5), although not to the same extent as those in the South. Consequently, the policy intensity regarding pollution control in the Midwest was marginally lower than that of the South. However, similar to the South, the Midwest has implemented more stringent policies over time and has successfully reduced emissions from stationary sources by more than half, from 45.31 million at the start of the 21^st^ century to 20.71 million in 2020 (5).

**A5. Fixed pollution of four regions**

**Table A1^*^.** Quantity of fixed pollution sources in four regions in 2020.

|  | Region | Midwest | Northeast | South | West |
| --- | --- | --- | --- | --- | --- |
| Industry | Beverages | 25 | 1 | 3 | 1 |
|  | Chemical Wholesalers | 451 | 210 | 621 | 209 |
|  | Chemicals | 3910 | 1069 | 5989 | 731 |
|  | Coal Mining | 17 | 0 | 19 | 17 |
|  | Computers and Electronic Products | 97 | 93 | 103 | 157 |
|  | Electric Utilities | 782 | 197 | 882 | 329 |
|  | Electrical Equipment | 207 | 96 | 269 | 54 |
|  | Fabricated Metals | 2001 | 745 | 1526 | 459 |
|  | Food | 598 | 62 | 318 | 86 |
|  | Furniture | 131 | 20 | 78 | 16 |
|  | Hazardous Waste | 698 | 133 | 701 | 318 |
|  | Leather | 14 | 0 | 3 | 0 |
|  | Machinery | 815 | 196 | 622 | 88 |
|  | Metal Mining | 28 | 4 | 41 | 395 |
|  | Miscellaneous Manufacturing | 119 | 74 | 82 | 36 |
|  | Nonmetallic Mineral Product | 723 | 298 | 1266 | 558 |
|  | Other | 80 | 53 | 303 | 183 |
|  | Paper | 302 | 200 | 908 | 159 |
|  | Petroleum | 734 | 338 | 1778 | 1123 |
|  | Petroleum Bulk Termi0ls | 486 | 526 | 1393 | 865 |
|  | Plastics and Rubber | 549 | 141 | 613 | 132 |
|  | Primary Metals | 1360 | 634 | 1044 | 302 |
|  | Printing | 46 | 20 | 27 | 7 |
|  | Textiles | 16 | 36 | 57 | 9 |
|  | Transportation Equipment | 1350 | 200 | 973 | 210 |
|  | Wood Products | 121 | 44 | 596 | 148 |
|  | Publishing | 0 | 1 | 0 | 0 |
|  | Textile Product | 0 | 3 | 21 | 0 |
|  | Apparel | 0 | 0 | 1 | 0 |
|  | Tobacco | 0 | 0 | 2 | 0 |

**Notes*: Data comes from the TRI program of EPA website.

**Fig. A5.** Emissions of total quantity of the toxic chemical from stationary facility of four regions.

**A6. MDS results for different parties, different regions vs federal government**

**Fig. A6.** MDS results of deep red states, deep blue states and the federal government.

**Fig. A7.** MDS results of four regions and the federal government.

**A7. Policy intensity comparison**

**Fig. A8.** Sum policy intensity of positive intensity, negative intensity, and total intensity policy.

# B. Data description and robust test

**B1. Descriptive data**

**Table B1.** Summary statistics of variables

| variable | Obs. | Mean | S.d. | Min. | Median | Max. |
| --- | --- | --- | --- | --- | --- | --- |
| Intensity | 599 | 20.57 | 63.57 | -32.86 | 5.280 | 583.6 |
| P_Intensity | 599 | 30.91 | 84.45 | 0.310 | 10.83 | 726.0 |
| N_Intensity | 599 | 10.34 | 23.40 | 0.0200 | 3.600 | 198.7 |
| avg_Intensity | 599 | 0.0200 | 0.0200 | -0.0500 | 0.0100 | 0.110 |
| P_avg_Intensity | 599 | 0.0300 | 0.0200 | 0 | 0.0300 | 0.170 |
| N_avg_Intensity | 599 | -0.0600 | 0.0400 | -0.350 | -0.0500 | -0.0100 |
| CO | 599 | 1439 | 1448 | 27.62 | 1129 | 9834 |
| NO_x_ | 599 | 251.4 | 231.1 | 4.190 | 187.7 | 1790 |
| PM_10_ | 599 | 359.1 | 314.6 | 3.560 | 297.2 | 2707 |
| PM_2.5_ | 599 | 111.7 | 101.7 | 0.980 | 87.64 | 742.4 |
| SO_2_ | 599 | 103.4 | 157.3 | 0.0600 | 48.70 | 1122 |
| GDP | 599 | 33.33 | 41.75 | 2.700 | 19.80 | 272.9 |
| wage | 599 | 90.33 | 12.21 | 0 | 92.34 | 99.41 |
| count | 599 | 98.34 | 12.31 | 0 | 100.1 | 100.4 |
| income | 599 | 29.86 | 37.31 | 1.730 | 18.23 | 279.1 |
| population | 599 | 631.6 | 716.6 | 56.45 | 449.2 | 3954 |
| CLTCB | 599 | 30.71 | 32.96 | 0 | 23.88 | 169.5 |
| CLTXD | 599 | 3.040 | 1.590 | 0 | 3.130 | 8.340 |
| CLTXV | 599 | 98.51 | 187.4 | 0 | 33.80 | 1464 |
| CO NA | 599 | 0.0800 | 0.560 | 0 | 0 | 8 |
| NO_x_ NA | 599 | 0 | 0 | 0 | 0 | 0 |
| PM_10_ NA | 599 | 0.720 | 1.910 | 0 | 0 | 16 |
| PM_2.5_ NA | 599 | 0.190 | 1.430 | 0 | 0 | 14 |
| SO2 NA | 599 | 0.620 | 1.280 | 0 | 0 | 6 |

**B2. Robust test of overall policy effect**

**Table B2.** Robust test using average intensity.

|  | (1) | (2) | (3) | (4) | (5) |
| --- | --- | --- | --- | --- | --- |
|  | CO | NO_x_ | PM_10_ | PM_2.5_ | SO_2_ |
| avgint | -2.58 | -0.33 | -0.83 | -0.23 | -0.38^*^ |
|  | (3.02) | (0.20) | (0.73) | (0.29) | (0.21) |
| CO NA | Y |  |  |  |  |
| NO_x_ NA |  | Y |  |  |  |
| PM_10_ NA |  |  | Y |  |  |
| PM_2.5_ NA |  |  |  | Y |  |
| SO_2_ NA |  |  |  |  | Y |
| CV | Y | Y | Y | Y | Y |
| State FE | Y | Y | Y | Y | Y |
| Party FE | Y | Y | Y | Y | Y |
| Year FE | Y | Y | Y | Y | Y |
| N | 599 | 599 | 599 | 599 | 599 |

*Notes:* * significant at 10% ** significant at 5% *** significant at 1%. CV refers to the control variables.

**Table B3.** Robust test using Intensity_t-1._

|  | (1) | (2) | (3) | (4) | (5) |
| --- | --- | --- | --- | --- | --- |
|  | CO | NO_x_ | PM_10_ | PM_2.5_ | SO_2_ |
| Intensity_t-1_ | -6.31^***^ | -0.21^***^ | -1.21^***^ | -0.59^***^ | -0.05 |
|  | (1.35) | (0.05) | (0.18) | (0.08) | (0.13) |
| CO NA | Y |  |  |  |  |
| NO_x_ NA |  | Y |  |  |  |
| PM_10_ NA |  |  | Y |  |  |
| PM_2.5_ NA |  |  |  | Y |  |
| SO_2_ NA |  |  |  |  | Y |
| CV | Y | Y | Y | Y | Y |
| State FE | Y | Y | Y | Y | Y |
| Party FE | Y | Y | Y | Y | Y |
| Year FE | Y | Y | Y | Y | Y |
| N | 458 | 458 | 458 | 458 | 458 |

*Notes:* * significant at 10% ** significant at 5% *** significant at 1%. CV refers to the control variables.

**Table B4.** Overall effect to different air pollutants

|  | (1) | (2) | (3) | (4) | (5) |
| --- | --- | --- | --- | --- | --- |
|  | CO | NO_x_ | PM_10_ | PM_2.5_ | SO_2_ |
| *Panel A* |  |  |  |  |  |
| Intensity | -8.49^***^ | -0.82^***^ | -1.00^***^ | -0.53^***^ | 0.16^**^ |
|  | (1.12) | (0.11) | (0.11) | (0.07) | (0.08) |
| State FE | Y | Y | Y | Y | Y |
| Party FE | Y | Y | Y | Y | Y |
| Year FE | Y | Y | Y | Y | Y |
| N | 599 | 599 | 599 | 599 | 599 |
|  |  |  |  |  |  |
| *Panel B* |  |  |  |  |  |
| Intensity | -8.51^***^ | -0.81^***^ | -1.00^***^ | -0.53^***^ | 0.17^**^ |
|  | (1.15) | (0.12) | (0.11) | (0.07) | (0.08) |
| wage | -13.86 | 1.21 | -1.22 | -1.64 | 3.11 |
|  | (23.60) | (3.44) | (4.00) | (1.54) | (3.77) |
| count | 83.77^*^ | 19.49^***^ | 7.59 | 1.62 | 22.18^**^ |
|  | (46.11) | (4.31) | (5.19) | (1.80) | (10.07) |
| State FE | Y | Y | Y | Y | Y |
| Party FE | Y | Y | Y | Y | Y |
| Year FE | Y | Y | Y | Y | Y |
| N | 599 | 599 | 599 | 599 | 599 |
|  |  |  |  |  |  |
| *Panel C* |  |  |  |  |  |
| Intensity | -6.63^***^ | 0.01 | -0.67 | -0.56^***^ | 0.67^***^ |
|  | (2.23) | (0.21) | (0.65) | (0.20) | (0.22) |
| wage | -22.20 | -1.25 | -1.74 | -1.69 | 0.90 |
|  | (19.54) | (2.33) | (3.81) | (1.53) | (2.53) |
| count | 31.11 | 4.77 | 4.50 | 1.18 | 8.13 |
|  | (34.55) | (3.40) | (11.14) | (2.86) | (5.18) |
| GDP | 51.61^**^ | 10.17^***^ | -13.93 | 0.56 | 11.70^***^ |
|  | (23.75) | (2.59) | (13.67) | (3.16) | (3.00) |
| income | -36.92^***^ | -9.81^***^ | 7.86 | -0.09 | -8.38^**^ |
|  | (13.36) | (2.50) | (6.64) | (1.60) | (3.19) |
| population | -2.84^***^ | -0.59^**^ | -0.21 | -0.05 | -0.79^***^ |
|  | (0.88) | (0.25) | (0.71) | (0.10) | (0.26) |
| State FE | Y | Y | Y | Y | Y |
| Party FE | Y | Y | Y | Y | Y |
| Year FE | Y | Y | Y | Y | Y |
| N | 599 | 599 | 599 | 599 | 599 |
|  |  |  |  |  |  |
| *Panel D* |  |  |  |  |  |
| Intensity | -7.81^***^ | -0.27^**^ | -1.07^**^ | -0.67^***^ | 0.07 |
|  | (2.03) | (0.13) | (0.53) | (0.18) | (0.12) |
| wage | -17.73 | -1.69 | -1.47 | -1.33 | 0.12 |
|  | (15.83) | (1.75) | (2.91) | (1.27) | (1.53) |
| count | 18.19 | 4.85 | 2.82 | 0.10 | 7.99 |
|  | (39.77) | (3.10) | (10.53) | (3.12) | (4.97) |
| GDP | 32.73 | 6.66^***^ | -19.57 | -1.22 | 4.09^**^ |
|  | (22.64) | (2.12) | (13.12) | (3.04) | (1.60) |
| income | -20.16 | -6.66^***^ | 12.97^*^ | 1.51 | -1.52 |
|  | (14.06) | (1.51) | (6.98) | (1.77) | (1.47) |
| population | -2.85^***^ | -0.51^***^ | -0.17 | -0.06 | -0.63^***^ |
|  | (1.02) | (0.16) | (0.63) | (0.10) | (0.20) |
| CLTCB | 16.81^***^ | 3.35^***^ | 4.70^***^ | 1.52^***^ | 7.07^***^ |
|  | (5.17) | (0.36) | (1.04) | (0.43) | (1.06) |
| CLTXD | 139.02 | -1.32 | 17.19 | 11.49 | 0.42 |
|  | (121.22) | (2.68) | (11.16) | (9.32) | (3.33) |
| CLTXV | -0.85^*^ | -0.03 | 0.03 | -0.05 | -0.02 |
|  | (0.43) | (0.04) | (0.10) | (0.03) | (0.07) |
| State FE | Y | Y | Y | Y | Y |
| Party FE | Y | Y | Y | Y | Y |
| Year FE | Y | Y | Y | Y | Y |
| N | 599 | 599 | 599 | 599 | 599 |

*Notes:* * significant at 10% ** significant at 5% *** significant at 1%.

**B3. Robust test of policy effect of positive and negative intensity**

**Table B5.** Robust test of positive intensity policy using average intensity

|  | (1) | (2) | (3) | (4) | (5) |
| --- | --- | --- | --- | --- | --- |
|  | CO | NO_x_ | PM_10_ | PM_2.5_ | SO_2_ |
| P_avg_Intensity | -7.82 | -0.35 | -1.06 | -0.56 | -0.09 |
|  | (6.49) | (0.37) | (1.27) | (0.53) | (0.38) |
| CO NA | Y |  |  |  |  |
| NO_x_ NA |  | Y |  |  |  |
| PM_10_ NA |  |  | Y |  |  |
| PM_2.5_ NA |  |  |  | Y |  |
| SO_2_ NA |  |  |  |  | Y |
| CV | Y | Y | Y | Y | Y |
| State FE | Y | Y | Y | Y | Y |
| Party FE | Y | Y | Y | Y | Y |
| Year FE | Y | Y | Y | Y | Y |
| N | 599 | 599 | 599 | 599 | 599 |

*Notes:* * significant at 10% ** significant at 5% *** significant at 1%. CV refers to the control variables.

**Table B6.** Robust test of positive intensity policy using Intensity_t-1_

|  | (1) | (2) | (3) | (4) | (5) |
| --- | --- | --- | --- | --- | --- |
|  | CO | NO_x_ | PM_10_ | PM_2.5_ | SO_2_ |
| P_Intensity_t-1_ | -4.97^***^ | -0.19^***^ | -0.96^***^ | -0.46^***^ | -0.02 |
|  | (1.18) | (0.05) | (0.17) | (0.07) | (0.11) |
| CO NA | Y |  |  |  |  |
| NOx NA |  | Y |  |  |  |
| PM_10_ NA |  |  | Y |  |  |
| PM_2.5_ NA |  |  |  | Y |  |
| SO_2_ NA |  |  |  |  | Y |
| CV | Y | Y | Y | Y | Y |
| State FE | Y | Y | Y | Y | Y |
| Party FE | Y | Y | Y | Y | Y |
| Year FE | Y | Y | Y | Y | Y |
| N | 458 | 458 | 458 | 458 | 458 |

*Notes:* * significant at 10% ** significant at 5% *** significant at 1%. CV refers to the control variables.

**Table B7.** Overall effect of positive intensity policy to different air pollutants

|  | (1) | (2) | (3) | (4) | (5) |
| --- | --- | --- | --- | --- | --- |
|  | CO | NO_x_ | PM_10_ | PM_2.5_ | SO_2_ |
| *Panel A* |  |  |  |  |  |
| P_Intensity | -7.67^***^ | -0.76^***^ | -0.90^***^ | -0.47^***^ | 0.16^**^ |
|  | (1.08) | (0.11) | (0.10) | (0.06) | (0.08) |
| State FE | Y | Y | Y | Y | Y |
| Party FE | Y | Y | Y | Y | Y |
| Year FE | Y | Y | Y | Y | Y |
| N | 599 | 599 | 599 | 599 | 599 |
|  |  |  |  |  |  |
|  |  |  |  |  |  |
| *Panel B* |  |  |  |  |  |
| P_Intensity | -7.68^***^ | -0.76^***^ | -0.90^***^ | -0.47^***^ | 0.17^**^ |
|  | (1.10) | (0.11) | (0.10) | (0.07) | (0.08) |
| wage | -12.03 | 1.36 | -1.01 | -1.52 | 3.08 |
|  | (23.52) | (3.43) | (3.99) | (1.53) | (3.77) |
| count | 83.96^*^ | 19.51^***^ | 7.62 | 1.63 | 22.18^**^ |
|  | (45.97) | (4.30) | (5.19) | (1.79) | (10.07) |
| State FE | Y | Y | Y | Y | Y |
| Party FE | Y | Y | Y | Y | Y |
| Year FE | Y | Y | Y | Y | Y |
| N | 599 | 599 | 599 | 599 | 599 |
|  |  |  |  |  |  |
|  |  |  |  |  |  |
| *Panel C* |  |  |  |  |  |
| P_Intensity | -5.73^***^ | -0.02 | -0.55 | -0.47^***^ | 0.62^***^ |
|  | (1.88) | (0.18) | (0.60) | (0.17) | (0.20) |
| wage | -20.93 | -1.25 | -1.61 | -1.58 | 0.77 |
|  | (19.54) | (2.33) | (3.78) | (1.53) | (2.53) |
| count | 31.37 | 4.81 | 4.48 | 1.19 | 8.04 |
|  | (34.50) | (3.41) | (11.11) | (2.83) | (5.14) |
| GDP | 51.60^**^ | 10.23^***^ | -14.02 | 0.54 | 11.58^***^ |
|  | (23.81) | (2.60) | (13.76) | (3.16) | (3.01) |
| income | -39.11^***^ | -9.76^***^ | 7.59 | -0.29 | -8.24^**^ |
|  | (13.71) | (2.49) | (6.71) | (1.67) | (3.18) |
| population | -2.60^***^ | -0.60^**^ | -0.18 | -0.03 | -0.80^***^ |
|  | (0.88) | (0.25) | (0.71) | (0.11) | (0.26) |
| State FE | Y | Y | Y | Y | Y |
| Party FE | Y | Y | Y | Y | Y |
| Year FE | Y | Y | Y | Y | Y |
| N | 599 | 599 | 599 | 599 | 599 |
|  |  |  |  |  |  |
|  |  |  |  |  |  |
| *Panel D* |  |  |  |  |  |
| P_Intensity | -6.70^***^ | -0.26^**^ | -0.89^*^ | -0.57^***^ | 0.11 |
|  | (1.69) | (0.12) | (0.49) | (0.15) | (0.09) |
| wage | -16.14 | -1.64 | -1.25 | -1.20 | 0.11 |
|  | (15.80) | (1.75) | (2.90) | (1.27) | (1.53) |
| count | 18.44 | 4.89 | 2.81 | 0.11 | 7.91 |
|  | (39.78) | (3.08) | (10.49) | (3.11) | (4.97) |
| GDP | 33.26 | 6.73^***^ | -19.55 | -1.19 | 3.99^**^ |
|  | (23.08) | (2.12) | (13.24) | (3.07) | (1.62) |
| income | -23.22 | -6.71^***^ | 12.48^*^ | 1.22 | -1.60 |
|  | (14.88) | (1.51) | (7.01) | (1.86) | (1.44) |
| population | -2.59^**^ | -0.51^***^ | -0.13 | -0.03 | -0.63^***^ |
|  | (1.06) | (0.16) | (0.62) | (0.11) | (0.19) |
| CLTCB | 16.10^***^ | 3.34^***^ | 4.59^***^ | 1.45^***^ | 7.05^***^ |
|  | (5.17) | (0.36) | (1.03) | (0.43) | (1.05) |
| CLTXD | 139.44 | -1.32 | 17.25 | 11.53 | 0.43 |
|  | (121.06) | (2.67) | (11.17) | (9.31) | (3.34) |
| CLTXV | -0.79^*^ | -0.03 | 0.04 | -0.04 | -0.02 |
|  | (0.43) | (0.04) | (0.10) | (0.03) | (0.07) |
| State FE | Y | Y | Y | Y | Y |
| Party FE | Y | Y | Y | Y | Y |
| Year FE | Y | Y | Y | Y | Y |
| N | 599 | 599 | 599 | 599 | 599 |

*Notes:* * significant at 10% ** significant at 5% *** significant at 1%.

**Table B8.** Robust test of negative intensity policy using average intensity

|  | (1) | (2) | (3) | (4) | (5) |
| --- | --- | --- | --- | --- | --- |
|  | CO | NO_x_ | PM_10_ | PM_2.5_ | SO_2_ |
| N_avg_Intensity | 0.19 | 0.13^**^ | 0.12 | 0.01 | 0.13^*^ |
|  | (0.82) | (0.05) | (0.20) | (0.07) | (0.07) |
| CO NA | Y |  |  |  |  |
| NO_x_ NA |  | Y |  |  |  |
| PM_10_ NA |  |  | Y |  |  |
| PM_2.5_ NA |  |  |  | Y |  |
| SO_2_ NA |  |  |  |  | Y |
| CV | Y | Y | Y | Y | Y |
| State FE | Y | Y | Y | Y | Y |
| Party FE | Y | Y | Y | Y | Y |
| Year FE | Y | Y | Y | Y | Y |
| N | 599 | 599 | 599 | 599 | 599 |

*Notes:* * significant at 10% ** significant at 5% *** significant at 1%.

**Table B9.** Robust test of negative intensity policy using Intensity_t-1_

|  | (1) | (2) | (3) | (4) | (5) |
| --- | --- | --- | --- | --- | --- |
|  | CO | NO_x_ | PM_10_ | PM_2.5_ | SO_2_ |
| N_Intensity_t-1_ | 6.11^*^ | -0.32 | 0.88 | 0.63^**^ | 0.43 |
|  | (3.48) | (0.32) | (0.74) | (0.31) | (0.41) |
| CO NA | Y |  |  |  |  |
| NO_x_ NA |  | Y |  |  |  |
| PM_10_ NA |  |  | Y |  |  |
| PM_2.5_ NA |  |  |  | Y |  |
| SO_2_ NA |  |  |  |  | Y |
| CV | Y | Y | Y | Y | Y |
| State FE | Y | Y | Y | Y | Y |
| Party FE | Y | Y | Y | Y | Y |
| Year FE | Y | Y | Y | Y | Y |
| N | 458 | 458 | 458 | 458 | 458 |

*Notes:* * significant at 10% ** significant at 5% *** significant at 1%.

**Table B10.** Overall effect of negative intensity policy to different air pollutants

|  | (1) | (2) | (3) | (4) | (5) |
| --- | --- | --- | --- | --- | --- |
|  | CO | NO_x_ | PM_10_ | PM_2.5_ | SO_2_ |
| *Panel A* |  |  |  |  |  |
| N_Intensity | -14.10 | -2.12 | -1.63 | -0.66 | 0.84 |
|  | (8.47) | (1.31) | (1.34) | (0.45) | (0.74) |
| State FE | Y | Y | Y | Y | Y |
| Party FE | Y | Y | Y | Y | Y |
| Year FE | Y | Y | Y | Y | Y |
| N | 599 | 599 | 599 | 599 | 599 |
|  |  |  |  |  |  |
|  |  |  |  |  |  |
| *Panel B* |  |  |  |  |  |
| N_intensity | -14.09 | -2.14 | -1.63 | -0.66 | 0.82 |
|  | (8.48) | (1.30) | (1.34) | (0.46) | (0.74) |
| wage | -2.08 | 2.42 | 0.16 | -0.93 | 2.81 |
|  | (25.06) | (3.73) | (4.03) | (1.52) | (3.72) |
| count | 85.31^*^ | 19.64^***^ | 7.77 | 1.71 | 22.15^**^ |
|  | (44.81) | (4.20) | (5.21) | (1.72) | (10.08) |
| State FE | Y | Y | Y | Y | Y |
| Party FE | Y | Y | Y | Y | Y |
| Year FE | Y | Y | Y | Y | Y |
| N | 599 | 599 | 599 | 599 | 599 |
|  |  |  |  |  |  |
|  |  |  |  |  |  |
| *Panel C* |  |  |  |  |  |
| N_intensity | -4.67 | -0.51 | 0.16 | -0.20 | 1.39^**^ |
|  | (4.42) | (0.60) | (1.58) | (0.33) | (0.60) |
| wage | -19.92 | -1.16 | -1.63 | -1.54 | 0.49 |
|  | (19.76) | (2.31) | (3.75) | (1.56) | (2.63) |
| count | 23.63 | 4.92 | 3.59 | 0.51 | 8.66^*^ |
|  | (32.89) | (3.38) | (10.68) | (2.58) | (5.00) |
| GDP | 37.56 | 10.39^***^ | -15.59 | -0.69 | 12.78^***^ |
|  | (23.10) | (2.59) | (12.60) | (2.91) | (3.31) |
| Income | -49.71^***^ | -9.83^***^ | 6.63 | -1.15 | -7.00^**^ |
|  | (18.51) | (2.31) | (7.45) | (2.07) | (3.17) |
| population | -1.19 | -0.59^**^ | -0.04 | 0.08 | -0.96^***^ |
|  | (0.98) | (0.23) | (0.73) | (0.12) | (0.25) |
| State FE | Y | Y | Y | Y | Y |
| Party FE | Y | Y | Y | Y | Y |
| Year FE | Y | Y | Y | Y | Y |
| N | 599 | 599 | 599 | 599 | 599 |
|  |  |  |  |  |  |
|  |  |  |  |  |  |
| *Panel D* |  |  |  |  |  |
| N_intensity | -5.11 | -0.67 | -0.17 | -0.27 | 1.00^**^ |
|  | (4.52) | (0.56) | (1.48) | (0.32) | (0.38) |
| wage | -14.53 | -1.49 | -1.13 | -1.09 | -0.09 |
|  | (16.46) | (1.74) | (3.00) | (1.36) | (1.54) |
| count | 9.61 | 4.67 | 1.51 | -0.67 | 7.83 |
|  | (39.02) | (3.00) | (10.06) | (2.95) | (4.85) |
| GDP | 21.83 | 6.46^***^ | -21.25 | -2.22 | 3.86^**^ |
|  | (25.60) | (1.92) | (12.78) | (3.11) | (1.75) |
| Income | -38.80^*^ | -7.34^***^ | 10.45 | -0.08 | -1.29 |
|  | (21.84) | (1.35) | (7.93) | (2.39) | (1.29) |
| population | -1.14 | -0.45^***^ | 0.06 | 0.09 | -0.65^***^ |
|  | (1.06) | (0.15) | (0.63) | (0.11) | (0.18) |
| CLTCB | 11.91^**^ | 3.18^***^ | 4.03^***^ | 1.10^**^ | 7.12^***^ |
|  | (5.55) | (0.34) | (1.25) | (0.49) | (1.01) |
| CLTXD | 141.54 | -1.23 | 17.53 | 11.71 | 0.38 |
|  | (121.10) | (2.66) | (11.19) | (9.32) | (3.40) |
| CLTXV | -0.74 | -0.02 | 0.05 | -0.04 | -0.03 |
|  | (0.45) | (0.04) | (0.11) | (0.04) | (0.07) |
| State FE | Y | Y | Y | Y | Y |
| Party FE | Y | Y | Y | Y | Y |
| Year FE | Y | Y | Y | Y | Y |
| N | 599 | 599 | 599 | 599 | 599 |

*Notes:* * significant at 10% ** significant at 5% *** significant at 1%.

# C. Materials and Methods

**C1. Policy basic features**

**Table C1.** Basic information of SIPs collection

| State | Dates of amendments | Number of policies |
| --- | --- | --- |
| Alabama | 2006,2009,2012-2020 | 11 |
| Alaska | 1993-2020 | 28 |
| Arizona | 2005,2010-2020 | 12 |
| Arkansas | 2010,2012,2014-2020 | 9 |
| California | 2005,2007,2009-2020 | 14 |
| Colorado | 2016-2020 | 5 |
| Connecticut | 2005,2009,2011,2012,2013,2014,2015,2016,2018,2019,2020 | 11 |
| Delaware | 2006, 2010,2011,2012,2014-2020 | 11 |
| District of Columbia | 2012-2020 | 9 |
| Florida | 1997-2020 | 24 |
| Georgia | 2006,2010,2013-2020 | 10 |
| Hawaii | 2009-2020 | 12 |
| Idaho | 2005,2010-2020 | 12 |
| Illinois | 2005,2010,2012-2020 | 11 |
| Indiana | 2006,2010-2020 | 12 |
| Iowa | 2009,2011,2013,2014-2020 | 10 |
| Kansas | 2006, 2009,2011-2020 | 12 |
| Kentucky | 2006,2009,2011-2020 | 12 |
| Louisiana | 2006,2009,2011-2020 | 12 |
| Maine | 2005,2009,2010-2020 | 13 |
| Maryland | 2005,2010,2013-2020 | 10 |
| Massachusetts | 2006,2009,2010,2012,2014-2020 | 11 |
| Michigan | 2006,2010,2011,2012,2014-2020 | 11 |
| Minnesota | 1997-2020 | 24 |
| Missouri | 2005,2009,2011-2020 | 12 |
| Mississippi | 2010,2012-2020 | 10 |
| Montana | 2005,2009,2011,2013,2014,2015,2017,2019,2020 | 9 |
| Nebraska | 2006,2009,2012-2020 | 11 |
| Nevada | 2005,2009,2010,2011,2013,2014,2015,2017,2019,2020 | 10 |
| New Hampshire | 2006,2009,2010,2012,2013-2020 | 12 |
| New Jersey | 2009,2013-2020 | 9 |
| New Mexico | 2006,2009,2011,2013-2020 | 11 |
| New York | 2006,2010,2012-2020 | 11 |
| North Carolina | 2005,2009-2016,2018,2019,2020 | 12 |
| North Dakota | 2009,2011-2020 | 11 |
| Ohio | 2006,2010-2020 | 12 |
| Oklahoma | 2006,2010,2012-2020 | 11 |
| Oregon | 2005,2007,2009,2011,2013,2015,2017,2019 | 8 |
| Pennsylvania | 2010,2012,2014-2020 | 9 |
| Rhode Island | 2005,2009,2010,2012-2020 | 12 |
| South Carolina | 2009,2010,2012-2020 | 11 |
| South Dokota | 2006,2010-2020 | 12 |
| Tennessee | 2010,2012,2014-2020 | 10 |
| Texas | 2005,2009,2011,2013,2015,2017,2019 | 7 |
| Utah | 2006,2010,2011,2012,2014-2020 | 11 |
| Vermont | 2005,2009,2011-2020 | 12 |
| Virginia | 2006,2010,2011,2013-2020 | 11 |
| Washington | 1973-1975,2002-2020 | 22 |
| West Virginia | 2005,2009-2020 | 13 |
| Wisconsin | 2010-2020 | 11 |
| Wyoming | 2010-2020 | 11 |

**Fig. C1.** Number and distribution of the State Implementation Plan. The line segment represents the time span over which the SIPs were issued. The solid line indicates that a state's policy formulation is continuous in the period, whereas the dotted line indicates that the state's policy promulgation is discontinuous in that period.

The disparities between states with regard to the cumulative number of policies implemented are depicted in Figure C1(A). Alaska, Minnesota, Florida and Washington exhibited the highest cumulative number of policies at 28, 25, 25 and 23, respectively. In contract, Colorado and Texas maintained the lowest cumulative numbers at 5 and 8. The temporal scope and continuity of policy enactments in each state are depicted in Figure C1(B), providing insight into the variability observed in the quantity of such enactments. The four states with the greatest number of policy-initiated policy enactment earlier and maintained policy enactment continuity. Alaska has been enacting state-level air control policies since 1993, followed by Florida, Minnesota and Washington. In contrast, the SIPs for Colorado became available post-2016, while those for Texas commenced in 2005 albeit with intermittent implementation. The existing variations in the efficacy of air pollution control across states may be partially accounted for by disparities in the timing and consistency of policy implementation.

**Fig. C2.** State average policy intensity between 2000-2020. States shaded with darker greens have a higher policy intensity, and states shaded with lighter greens have a weaker policy intensity. Figure A represents the year before 2000, Figure B represents the year between 2001-2007, Figure C represents the year between 2008-2014, and Figure D represents the year between 2015-2020.

**C2. Text preprocessing of policy documents**

Policy intensity has emerged as a powerful tool for quantitative policy research. It’s an index reflecting the stringency and importance of policy (6, 7), which is also regarded as “importance”, “stringency” and some other concepts. Many international organizations, such as the OECD, also define the policy stringency and measure 13 policy instruments for 40 countries (8, 9). Policy intensity indicators have progressively supplanted proxy indicators in policy analysis to compensate for the inherent risk of irrelevant factors influencing policy analysis and the inadequate representation of indicator that proxy indicators entail.

Measurement of policy intensity were most based on coding of policy contents, such as dividing policies into intensity levels and intensity scope (10), the national administrative power structure and the ranking of the policy type (11), the Index of Policy Activity (IPA) (12) and so on. However, the approach to encoding policies places significant burdens on the coders, encompassing their physical and mental well-being, comprehension of the policy, and skill level. The prevalence of this issue will escalate in tandem with the expansion of policy numbers and the intricacy of policy frameworks.

The natural language processing (NLP) method used in this paper is a further improvement to the method of policy contents analysis in the past, which provides an alternative to quantify the stringency of policy from a new perspective, and this research is to assess the validity of the proposed measure. NLP uses machine to substitute artificial quantification to quantify policies in sentences, and gets two indicators of polarity and subjectivity for each policy in the sentence dimension.

For polarity in the range of (-1,1), the closer the positive score to 1 signifies a stronger substantial support, approval and encouragement, suggesting that particular actions or behaviors should be promoted or enhanced. Conversely, the closer the negative score to -1 signifies stronger opposition, denial, and prohibition, suggesting that certain actions or behaviors should be prohibited or restricted. In the range of (0,1), the subjectivity score signifies the extent to which the government is concerned, attentive or proactive with regard to air pollution concerns. The expression becomes more robust as the score increases. Referring to previous studies of policy intensity (13, 14), we construct the following indicator:

$$\begin{aligned} intensity=polarity\times subjectivity \#\left( 1 \right) \end{aligned}$$

As mentioned above, policy intensity can be regarded as a comprehensive indicator of policy direction and importance. The following five example sentences from the 1990 Amendment of the CAA demonstrate the meaning of the indicators constructed in this study.

**Table C2.** Policy text indicators of 1990 Amendments of the Clean Air Act.

| Sentence | Polarity | Subjectivity | Intensity |
| --- | --- | --- | --- |
| 1."Whenever the Governor of a State has submitted a notice under clause (iv), the Governor, in consultation with State and local air pollution control agencies, shall undertake a study to evaluate whether the entire metropolitan statistical area or consolidated metropolitan statistical area should be included within the nonattainment area. " | 0 | 0.3125 | 0 |
| 2."The regulations shall contain standards for such fuels or vehicles, or both, which the Administrator determines reflect the greatest degree of emission reduction achievable through the application of technology which will be available, taking into consideration the standards established under subsection (a), the availability and costs of the technology, and noise, energy, and safety factors, and lead time. " | 0.47 | 0.63 | 0.2916 |
| 3."The Governor of any State may, on the Governor's own motion, submit to the Administrator a revised designation of any area or portion thereof within the State." | 0.6 | 1 | 0.6 |
| 4."Any such conditional approval shall be treated as a disapproval if the State fails to comply with such commitment." | -0.17 | 0.43 | -0.0731 |
| 5."falsifies, tampers with, renders inaccurate, or fails to install any monitoring device or method required to be maintained or followed under this Act shall, upon conviction, be punished by a fine pursuant to title 18 of the United States Code, or by imprisonment for not more than 2 years, or both. " | -0.11 | 0.43 | -0.0473 |

For instance, sentence #1 impartial depiction of the duty that state governors have to evaluate regional compliance, refraining from endorsing or proscription of any particular conduct. Polarity and intensity are therefore both zero. Sentence #2 delineates the standards that comprise regulations and the anticipated outcomes associated with them, whereas sentence #3 delineates the authority of governors to amend laws and regulations in any jurisdiction within their states. Although both sentences exhibit positive polarity, sentence #3 is a more forceful in its intensity due to the fact that the federal government has enhanced state support and empowerment. Sentence #4 describes the situation in which states will have their applications blocked if they fail to fulfill their obligations, sentence #5 outlined the penalties that states will incur if they violate the CAA’s requirements. Both provide accounts of the prohibition and punishment of actions carried out by the federal government, exhibiting a comparable degree of negative polarity. The aforementioned instances demonstrate that the NLP toolkit is capable of computing and distinguishing the intensity and direction of policy instruments.

Differences in the selection of policy instruments by states can also be reflected in the distribution of average policy intensity across states. In general, the implementation of more progressive, encouraging, and positive policy instruments aids in the modernization of the states' industrial structures and the attainment of goals that integrate environmental protection and economic development.

As illustrated in Figure 2, although Kentucky, Ohio, and Pennsylvania, all of which are situated in the Rust Belt, are subject to more intense transitional pressures, their average policy intensity is considerably higher than that of the remaining states. This can be primarily attributed to the greater policy polarity exhibited by all three states (as illustrated in SI Appendix, Fig.A1), which signifies a propensity for employing policy instruments that are more liberal, constructive, and encouraging.

(1) **Kentucky**: In the SIP issued by Kentucky in 2011, for instance, a significant proportion (30 percent) of policy texts exhibit a polarity exceeding 0.1, in comparison to the polarity of texts issued by other states. Upon closer examination of all policy statements in Kentucky with a magnitude greater than 0.1, it becomes evident that the state primarily regulates air pollution through the delegation of authority to local governments, the simplification of procedures, and the promotion of public participation.

Kentucky has implemented measures to streamline and optimize the approval process for air pollution sources, licensing, and approval processes. These measures aim to enhance the efficiency of approvals and foster a more permissive governance environment for the state's air pollution management.

- Example 1: “A person registering a motor vehicle in a county required to conduct vehicle emission testing under this chapter, who is transferring the registration of the vehicle from another state that is also required to conduct vehicle emission testing by the Federal Environmental Protection Agency, shall be issued a reciprocal certificate upon presenting to the cabinet or a county, air pollution control district, contractor, or fleet operator authorized by the cabinet, a valid certificate issued by the other state indicating the vehicle was tested and either successfully passed the inspection or was exempt from the inspection.” -- By issuing reciprocal certificates to vehicles tested in other states, Kentucky has simplified vehicle approval procedures and also conveyed confidence and support for air pollution management in other states.
- Example 2: “The air pollution control district shall, by regulation, allow changes within a permitted source or one operating pursuant to a timely application under subsection of this section, without requiring a permit revision, if the changes are not modifications under any provision of Title I of the Federal Clean Air Act of 1963 as amended by the Clean Air Act Amendments of 1990, and if the changes do not exceed the emissions allowable under the permit, whether expressed as a rate of emissions or in terms of total emission.” -- The requirement for modification of licenses is more liberal, and it is also a simplification of administrative approval procedures.

In the aspect of local authority, Kentucky fully empowers the local authorities in terms of the use of funds, administrative approvals, etc. and made it more flexible in air governance.

- Example 1: “The county may use the county’s emission inspection fund to acquire any special equipment, tools, employees, material, or facilities needed to adequately administer, investigate, or enforce the provisions of KRS 224.20-710 to 224.20-765.” -- States have been fully empowered to use the fund to purchase emission reduction materials.
- Example 2: “The air pollution control board may provide by regulation a schedule of annual emission fees to cover all reasonable direct and indirect costs required to maintain authorization to develop and administer the district’s permit program requirements under Title V of the Federal Clean Air Act of 1963 as amended by the Clean Air Act Amendments of 1990.” -- Administrative staff can adjust their plans for paying annual emissions fees according to the realities of the region.

In support of public involvement, Kentucky fully encourages public participation in the air pollution management process in its policy text.

- Example 1: “These rules shall provide the public with a fair and reasonable opportunity for review of and comment on all proposed actions on regulations and shall ensure that the board provides full consideration to all written and oral comments prior to promulgating final regulations.” -- The emphasis was placed on encouraging public intervention and the full consideration of public opinion in the enactment of the final legislation.
- Example 2: “The record of the hearing shall be open to public inspection, and copies thereof shall be made available to a person upon payment of the actual cost of reproducing the original, except as otherwise provided in district regulations.” -- It is a sign of Kentucky 's openness to the public to the pollution management process and the public's commitment to participate in the management of air pollution.

(2) **Ohio**: Take the SIP announced in 2011 in Ohio, where the proportion of policy polarity that are more than 0.1 is approximately 29%. In contrast to Kentucky, the policy text in Ohio places significant emphasis on the promotion of public participation and the delegation of administrative authority (polarity greater than 0.1). However, in Ohio, the executive is endowed with authority that imposes specific restrictions on its scope and limitations, and the incentive for public participation is comparatively meager in comparison to Kentucky.

Ohio's delegation of executive powers is more stringent with regard to administrative empowerment compared to Kentucky's. The executive is obligated to exercise power within specific limitations or prerequisites.

- Example 1: “The director may consider any significant departure from the operations of the source described in the permit to install application that results in greater emissions than the emissions rate modeled to determine the ground level concentration as a modification and require the owner or operator to submit a permit to install application for the increased emissions.” -- The directors may impose operational constraints on owners or operators in the event of significant deviations in emissions.
- Example 2: “In requiring monitoring devices, records, and reports, the director, to the extent consistent with the federal Clean Air Act, shall give consideration to technical feasibility and economics reasonableness and allow reasonable time for compliance.” -- This requires the administration to take administrative measures with due regard to external conditions.

Secondly, in encouraging public participation, Ohio imposes certain provisions and restrictions on groups involved in air pollution management.

- Example 1: “The Ohio air quality development authority shall conduct outreach activities in Ohio that seek to include minorities in the grant and loan program for advanced energy projects established under section 166.30 of the Revised Code.” -- This takes into account the right to guarantee minority participation in the improvement of air quality.

(3) **Pennsylvania**: Regarding the 2012 SIP implemented by Pennsylvania, approximately 48% of policies in the policy text have a policy intensity greater than 0.1. However, in contrast to Kentucky and Ohio, the active policy tools utilized in Pennsylvania are primarily concerned with elucidating the accountability and jurisdiction of those accountable for air pollution emissions.

- Example 1: “Costs established as recoverable under paragraph (2) shall qualify as nonrevenue-producing investment to improve environmental conditions under section 1315 (relating to limitation on consideration of certain costs for electric utilities), provided that any benefits to the utility generated by the sale of allowances under the Clean Air Act shall be flowed through to the utility’s ratepayers.” -- It emphasizes how the interests of the emitters are allocated on the basis of compliance with the CAA requirements.
- Example 2: “The utility shall not be required to refile its plan or to seek additional commission approvals concerning its plan unless the utility’s plan is significantly amended or revised.” -- This simplifies the procedures for administrative approval of emission subjects.
- Example 3: “The public utility shall make available, upon request, a copy of the proposed plan to any coal supplier with which it has a supply contract for more than one year and to any collective bargaining representative for the coal supplier.” -- This illustrates the responsibilities of utility companies in cooperation with air pollution entities.

Consequently, positive, encouraging, and liberal policies have additionally contributed to the facilitation of the transition processes in Pennsylvania, Ohio and Kentucky. Despite the fact that Kentucky, Ohio, and Pennsylvania are all situated within the Rust Belt, all three have led the transition process with industries that are also more successful in the Rust Belt. An assembly of numerous robotics research centers and corporations, including Aethon Inc., the National Robotics Engineering Center, and the Robotics Institute, was observed in Pittsburgh, Pennsylvania. Formerly known as the "Rubber Capital of the World," Akron, Ohio is now widely recognized as a hub for polymer research, a partnership between the city mayor's office and the University of Akron, utilizing the city's rubber industry foundation (15). Additive manufacturing, also known as 3D printing, presents an additional auspicious pathway for the revival of manufacturing. Using 3-D imaging systems, businesses such as MakerGear of Beachwood, Ohio, and ExOne Company of North Huntingdon, Pennsylvania, design and manufacture industrial and consumer goods (16). The three above examples demonstrate that more proactive and motivating air pollution policies can the objectives of air pollutant management without affecting economic development, and that this is undoubtedly a good basis for the transition of other Rust Belt states to combining air environmental pollution management with economic development.

**Table C3.** Steps of analyzing the U.S. CAA and SIPs

| Stage | Step | Approach |
| --- | --- | --- |
| S1.Split | 1.sentence segmentation | Split the policy document into individual sentences |
| S2.Calculation | 2.sentiment calculation | Calculate the length, polarity and subjectivity of each sentence |
|  | 3.intensity calculation | Calculate the intensity of each sentence based on Formula 1 |
| S3.Aggregation | 4.document-level aggregation | Aggregate the sentence-level scores (polarity, subjectivity, and intensity) at the document level by calculating average and sum scores |
| S4.Analysis | 5.multidimensional analysis | Aggregate and compare the policy intensity scores based on the dimensions of year, level (federal vs. state), political polarization (red vs. blue), and region (Northeast, Midwest, South, West). |

**Table C4.** The meaning of the corresponding parameters.

| Parameter | Meaning |
| --- | --- |
| ${\hat{\Pi}'}_{X_{T}}$ and ${\hat{\Pi}'}_{Y_{T}}$ | The parameter estimations of $X_{T}$ and $Y_{T}$ in$AR(k)$ |
| k | Piccolo's distance^48^ |
| $\hat{V}$ | The parameter estimation of $V=\sigma_{X_{T}}^{2}R_{X_{T}}^{-1}\left( k \right)+\sigma_{Y_{T}}^{2}R_{Y_{T}}^{-1}\left( k \right)$ |
| $\sigma_{X_{T}}^{2}$ and $\sigma_{Y_{T}}^{2}$ | The variance in white noise associated with $X_{T}$ and $Y_{T}$, respectively |
| $R_{X_{T}}$ and $R_{Y_{T}}$ | The sample covariance matrix of two sequences |

**Table C5.** The list of variables and corresponding meaning.

| Variable | Specific variable | Meaning |
| --- | --- | --- |
| ${Pollute}_{ijt}$ |  | Emissions of air pollutant $j$ in state $i$, year $t$ |
| ${Intensity}_{it}$ |  | SIP intensity in state $i$, year $t$ |
| $X_{it}$ | ${GDP}_{it}$ | Actual GDP in state $i$, year $t$ |
|  | ${population}_{it}$ | Total population in state $i$, year $t$ |
|  | ${income}_{it}$ | Personal income in state $i$, year $t$ |
|  | ${count}_{it}$ | The number of industrial establishments in state $i$, year $t$ |
|  | ${wage}_{it}$ | The average industrial wage in state $i$, year $t$ |
|  | ${CLTCB}_{it}$ | The total coal consumption in state $i$, year $t$ |
|  | ${CLTXD}_{it}$ | The end-use coal price in state $i$, year $t$ |
|  | ${CLTXV}_{it}$ | The total end-use coal expenditure in state $i$, year $t$ |
|  | ${Pollute}_{ijt} NA$ | The number of non-attainment area in state $i$, year $t$ |

# SI References

1. EPA (1995) EPA Commits to Partnership for Long-Term Research on Remote-Sensing Data from Space Satellites. (EPA press release).
2. A. C. Stern, History of air pollution legislation in the United States. *J Air Pollut Control Assoc* **32**, 44-61 (1982).
3. R. Schmalensee, R. Stavins, Policy Evolution under the Clean Air Act. *Journal of Economic Perspectives* **33**, 27-50 (2019).
4. K. W. Appel *et al.* (2013) Application and evaluation of the two-way coupled WRF-CMAQ modeling system to the 2011 DISCOVER-AQ campaign in the Baltimore-Washington D.C. area. in *12th Annual CMAS Conference* (EPA, Chepel Hill, NC).
5. EPA, TRI Basic Data Files: Calender Years 1987-Present. https://www.epa.gov/toxics-release-inventory-tri-program/tri-basic-data-files-calendar-years-1987-present.
6. B. Cashore, M. Howlett, Punctuating Which Equilibrium? Understanding Thermostatic Policy Dynamics in Pacific Northwest Forestry. *American Journal of Political Science* **51**, 532-551 (2007).
7. M. Howlett, Governance modes, policy regimes and operational plans: A multi-level nested model of policy instrument choice and policy design. *Policy Sciences* **42**, 73-89 (2009).
8. T. Kruse, A. Dechezleprêtre, R. Saffar, L. Robert, Measuring environmental policy stringency in OECD countries. doi:https://doi.org/10.1787/90ab82e8-en (2022).
9. A. Fabrizi, G. Guarini, V. Meliciani, Green patents, regulatory policies and research network policies. *Research Policy* **47**, 1018-1031 (2018).
10. C. Knill, K. Schulze, J. Tosun, Regulatory policy outputs and impacts: Exploring a complex relationship. *Regulation & Governance* **6**, 427-444 (2012).
11. L. Liu, T. Zhang, A.-P. Avrin, X. Wang, Is China's industrial policy effective? An empirical study of the new energy vehicles industry. *Technology in Society* **63** (2020).
12. T. S. Schmidt, S. Sewerin, Measuring the temporal dynamics of policy mixes – An empirical analysis of renewable energy policy mixes’ balance and design features in nine countries. *Research Policy* **48** (2019).
13. G. Zhang, N. Deng, H. Mou, Z. Zhang, X. Chen, The impact of the policy and behavior of public participation on environmental governance performance: Empirical analysis based on provincial panel data in China. *Energy Policy* **129**, 1347-1354 (2019).
14. G. Zhang *et al.*, China’s environmental policy intensity for 1978–2019. *Scientific Data* **9**, 75 (2022).
15. S. Karabin, Mayor says attitude is key to Akron’s revitalization. *Akron legal news* https://www.akronlegalnews.com/editorial/7004 (2013).
16. L. Boselovic, Conference in Pittsburgh shows growing allure of 3-D printing. *Pittsburge Post-Gazette* https://www.post-gazette.com/business/businessnews/2013/06/13/Conference-in-Pittsburgh-shows-growing-allure-of-3-D-printing/stories/201306130342 (2013).
